# Investigation on LASSBio‐1971 and LASSBio‐1974 Cellular Cytotoxic Mechanism and Their Comparative DMPK Profile

**DOI:** 10.1002/ardp.70151

**Published:** 2025-11-21

**Authors:** Manoel Oliveira de Moraes Junior, Gisele Barbosa, Caroline Marques Xavier da Costa, Raysa Magali Pillpe‐Meza, Wesley Leandro de Gouveia, Daniel Nascimento do Amaral, Luis Gabriel Valdivieso Gelves, Stefan Laufer, Lídia Moreira Lima

**Affiliations:** ^1^ National Institute of Science and Technology of Pharmaceuticals and Medications (INCT‐INOFAR) Federal University of Rio de Janeiro, Laboratory of Evaluation and Synthesis of Bioactive Substances (LASSBio), CCS, University City Rio de Janeiro Brazil; ^2^ Postgraduate Program in Pharmacology and Medicinal Chemistry, Institute of Biomedical Sciences Federal University of Rio de Janeiro Rio de Janeiro Brazil; ^3^ Department of Pharmaceutical Chemistry IPS, Eberhard Karls Universität Tübingen Tübingen Germany

**Keywords:** DMPK, EGFR inhibitor, EGFR resistance, glutathione‐adduct, non‐small cell lung cancer

## Abstract

Due to the arising of clinically relevant resistant EGFR‐related phenotype through innovative mechanisms, mainly EGFR_L858R/T790M_, the emergence of novel molecules with dual or multi‐target affinity has presented a promising alternative to overcoming these resistance mechanisms. This study aimed to evaluate synthetic acrylamide quinoxaline derivatives against NSCLC cell lines with different overexpressed EGFR mutations and compare their DMPK profile. The biological activity of LASSBio‐1971 and LASSBio‐1974 was assessed through cytotoxicity (MTT and Sulforhodamine B assays), apoptosis induction, EGFR inhibition, cell cycle analysis (flow cytometry), immunofluorescence microscopy, cell membrane permeability (PAMPA assay), and metabolic stability in rat liver microsomes. LASSBio‐1971 exhibited promising EGFR inhibition with favorable in vitro pharmacokinetic (PK) properties, including high gastrointestinal and blood–brain barrier permeability. LASSBio‐1974 demonstrated nonselective mechanism inhibiting EGFR and mitotic machinery leading to apoptosis and cell cycle arrest at different phases. LASSBio‐1971 and LASSBio‐1974 emerge as EGFR inhibitors with equipotent cytotoxic effects on human NSCLC lines and different in PK profile. Further studies should be conducted with LASSBio‐1974 to prove and understand its antimicrotubule action.

## Graphical abstract

The EGFR covalent inhibitors **1** and **2** were evaluated in phenotypic assays. Although they displayed good cytotoxic potency on human NSCLC lines containing different expressions of the EGFR wild‐type and mutant forms, the first exhibited the most favorable in vitro pharmacokinetic profile, while the latter revealed anti‐EGFR and anti‐microtubule actions.

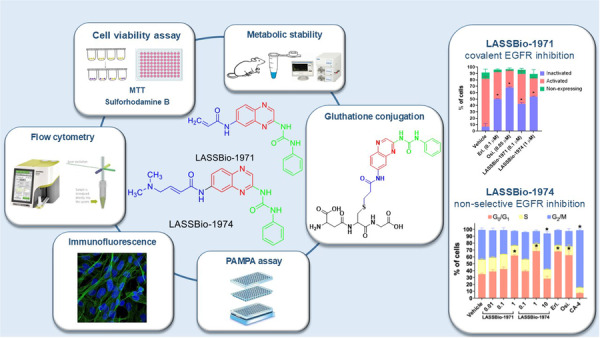

## 1 Introduction

Lung cancer has been one of the most prevalent and deadliest cancer types across the world, with about 2.5 million new cases and 1.8 million deaths in 2022, projecting 3.55 million deaths in 2050 [1, 2]. In about 80% of all cases, the most common type is known as non‐small cell lung cancer (NSCLC), classified based on morphophysiological cell characteristics [3, 4]. Beyond the classic cytotoxic chemotherapy drugs available, epidermal growth factor receptor (EGFR) inhibitors (EGFRi) have been consolidated as the most important class of targeted therapy against NSCLC [5, 6, 7].

EGFRs are transmembrane tyrosine kinase proteins, members of the ERBb family along with HER‐2, HER‐3, and HER‐4, which are present in epidermal cells, mostly activated by the epidermal growth factor (EGF) [8, 9]. Once the complex EGF‐EGFR is formed, it can dimerize and auto‐phosphorylate to activate signaling pathways including MAPK, PI3K‐AKT‐mTOR, and JAK‐STAT, all associated with proliferation, survival, angiogenesis, migration, and antiapoptotic phenotypes [10, 11]. Deregulation of the EGFR system is related to the development of NSCLC with poor prognosis. Thus, inhibiting overexpressed EGFR in aberrant NSCLC cells has been shown to be a powerful anticancer mechanism of action [12, 13, 14].

**Figure 1:**
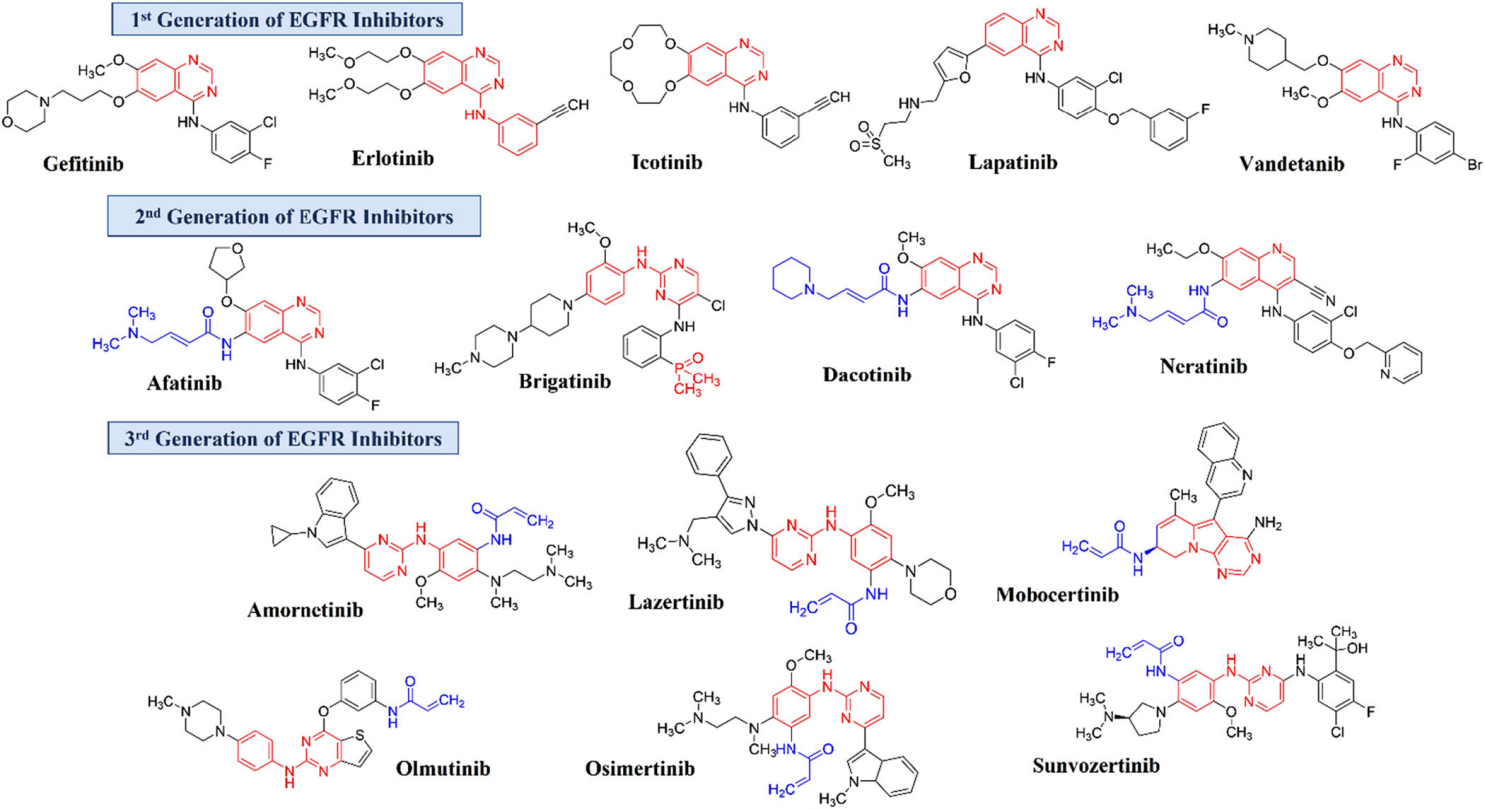
Chemical structure of EGFR Inhibitors: The rigid structure (scaffold) is highlighted in red and the electrophilic groups in blue.

Regardless of the improvement in NSCLC therapy success with EGFRi molecules, the appearance of EGFR point mutations has been connected to changes in adequate responsiveness. The first generation of EGFRi (Figure 1), usually represented by erlotinib and gefitinib, was developed to inhibit wild‐type EGFR, and yet the L858R point mutation (replacement of leucine to arginine at 858‐position in exon 21) was later related to better treatment results due to a decrease in ATP‐EGFR_L858R_ interaction affinity in its catalytic binding site [15, 16, 17].

On the other hand, resistance phenotypes can also arise, as in the case of double mutation L858R/T790M (L858R plus replacement of threonine to methionine at 790‐position in exon 20), which increases ATP‐EGFR_T790M_ binding affinity kinetics. Nonetheless, most of those initially good responsive patients display later resistance issues associated with the T790M mutation occurrence [18, 19, 20].

Second‐generation EGFR inhibitors (Figure 1), represented by afatinib, inaugurated the use of covalent drug‐EGFR interaction strategy to overcome the increased ATP‐EGFR_L858R_ affinity, resulting in greater potency but off‐target activity [21, 22, 23]. Although this off‐target interaction did not solve the problem of therapeutic resistance related to double‐mutated EGFR, it has proved to be beneficial by inhibiting EGFR and HER‐2, a crucial aberrant receptor of other types of tumors such as breast cancer [24, 25].

Later, the third generation of EGFRi (Figure 1) selective to EGFR_L858R/T790M_ was developed (e.g., osimertinib) and seemed to help as an alternative against NSCLC patients resistant to the first generation EGFR‐inhibitor [26, 27, 28]. However, other resistant phenotypes have emerged, associated with or not EGFR‐mutated progression such as EGFR_L858R/T790M/C797S_, bypass mechanisms, or epithelial–mesenchymal transition [19, 20, 29, 30, 31, 32].

Therefore, it is worthy strive to develop new molecules harboring the capacity to selectively inhibit the clinically relevant resistant EGFRL858R/T790M with brand new mechanisms. Recently, besides the EGFR inhibition mechanism, the emergence of novel molecules with dual or multi‐target affinity has been shown as an alternative to overcome these resistance mechanisms [33, 34, 35].

**Figure 2:**
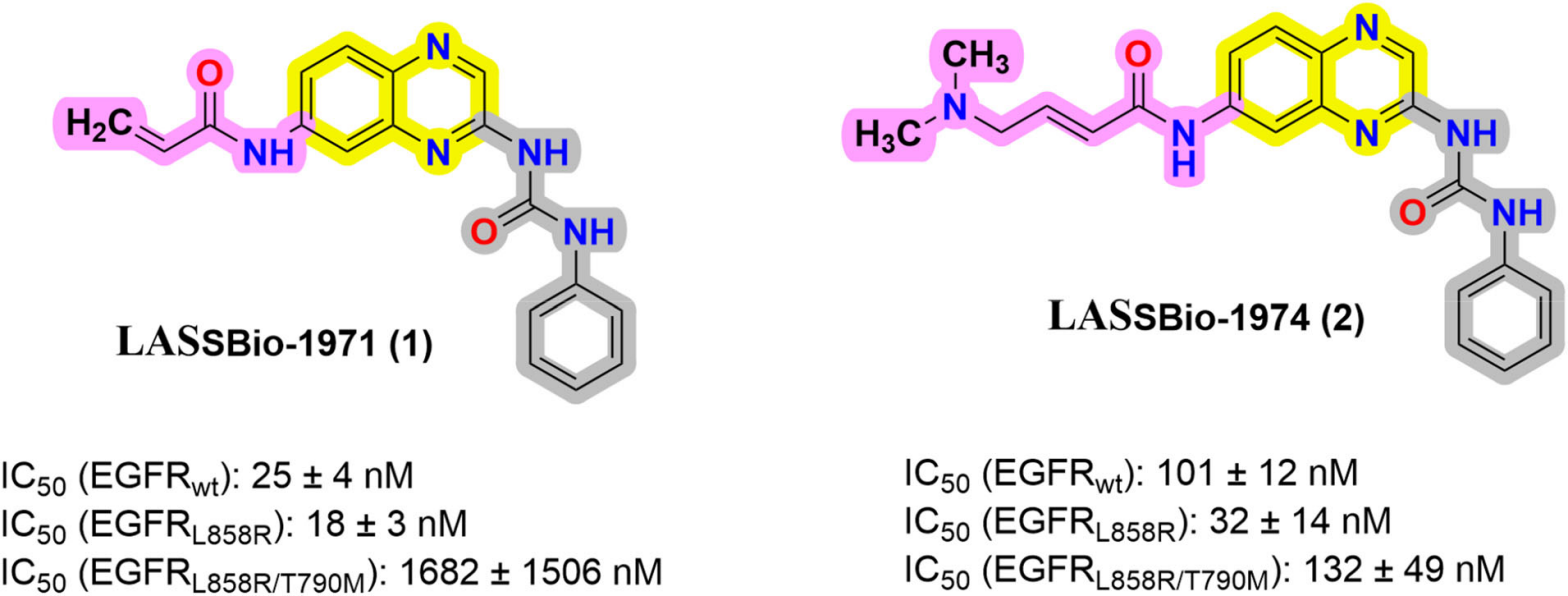
Chemical structures of EGFR inhibitors LASSBio‐1971 (**1**) and LASSBio‐1974 (**2**) and their potency against EGFR wild‐type and mutant forms [36].

In this context, our research group has previously described the design, synthesis, and in vitro potential activity of brand‐new acrylamide‐quinoxaline derivatives as a novel scaffold for EGFR inhibition [36]. On this basis, the present work focuses on the evaluation of two synthetic acrylamide quinoxaline derivates LASSBio‐1971 (**1**) and LASSBio‐1974 (**2**) (Figure 2) to study, in phenotypic assays, their comparative cytotoxicity against NSCLC cell lines, apoptosis induction, EGFR inhibition, cell cycle behavior by flow cytometry, morphology by immunofluorescence microscopy, cell membrane permeability by PAMPA assay, and metabolic stability in rat liver microsomes (RLMs).

## 2 Results

### 2.1 Cell Viability

The cytotoxic potential of LASSBio‐1971 (**1**) and LASSBio‐1974 (**2**) (Figure 2) was evaluated by MTT and sulforhodamine B (SRB) techniques comparing with the FDA‐approved EGFR‐inhibitors, erlotinib (first generation) and osimertinib (third generation). LASSBio‐1971 (**1**) showed nanomolar activity against PC‐9_L858R_ (0.13 µM) measured by MTT and a low micromolar value (1.02 µM) by SRB (Tables 1 and 2, Figure S1). Considering the EGFR_wt_ NCI‐H292 and EGFR double‐resistant NCI‐H1975_L858R/T790M_ cell lines, their half‐maximal cytotoxic concentration (CC_50_) value ranged on a micromolar scale, 1.5/4.95 µM for NCI‐H292, and 5.1/18.47 µM for NCI‐H1975, both via MTT and SRB, respectively (Tables 1 and 2, Figure S1). These results can also express their activity on less sensitive cells, such as NCI‐H1975.

**Table 1:** LASSBio‐1971 (**1**) and LASSBio‐1974 (**2**) cytotoxic activity (CC_50_, µM) against NSCLC cell lines in a 72‐h period assessed by MTT assay.

Compounds	NCI‐H292 (EGFR_wt_)	PC‐9 (EGFR_L858R_)	NCI‐H1975 (EGFR_L858R/T790M_)
LASSBio‐1971 (**1**)	1.51	0.13	5.1
	(0.9–2.52)	(0.07–0.26)	(3.78–6.9)
LASSBio‐1974 (**2**)	4.98	1.1	7.09
	(3.59–6.91)	(0.7–1.73)	(5.74–8.76)
Erlotinib	15.31	0.1	21.05
	(9.78–23.96)	(0.04–0.23)	(14.15–31.31)
Osimertinib	6.3	0.05	0.1
	(5.22–7.6)	(0.02–0.12)	(0.05–0.2)

**Table 2:** LASSBio‐1971 (**1**) and LASSBio‐1974 (**2**) cytotoxic activity (CC_50_, µM) against NSCLC cell lines in a 72‐h period assessed by SRB assay.

Compounds	NCI‐H292 (EGFR_wt_)	PC‐9 (EGFR_L858R_)	NCI‐H1975 (EGFR_L858R/T790M_)
LASSBio‐1971 (**1**)	4.95	1.02	18.47
	(4.01–5.99)	(0.57–1.83)	(14.01–24.35)
LASSBio‐1974 (**2**)	8.99	1.22	20.51
	(5.72–14.14)	(0.61–2.43)	(15.12–27.82)
Erlotinib	39.82	0.2	9.69
	(36.94–42.92)	(0.06–0.67)	(6.58–14.27)
Osimertinib	6.86	0.07	0.14
	(2.04–23.07)	(0.04–0.12)	(0.07–0.28)

On the other hand, LASSBio‐1974 (**2**) showed cytotoxicity in a micromolar range for all NSCLC cells (Tables 1 and 2, Figure S1). This compound demonstrated better cytotoxic activity against PC‐9L_8585R_ (CC_50_ 1.1/1.22, MTT and SRB, respectively). Additionally, NCI‐H292 and NCI‐H1975 with different EGFR mutations were also LASSBio1974‐sensible. Erlotinib and osimertinib EGFR‐inhibitors also showed more cytotoxic against PC‐9_L8585R_ as expected. Moreover, only osimertinib demonstrated nanomolar CC_50_ against NCI‐H1975_L858R/T790M_.

Comparing CC_50_ values from MTT and SRB methods, all compounds showed very similar outcomes evidencing their reproducibility potential and cytotoxic activity throughout different cell viability parameters, such as mitochondrial metabolism activity (MTT) along with a cellular protein mass measurement (SRB) representing proliferative activity. LASSBio‐1971 (**1**) against PC‐9_L8585R_ was the only result to oscillate from nano to a micromolar scale within about a 10‐fold change.

### 2.2 Apoptosis Evaluation

**Figure 3:**
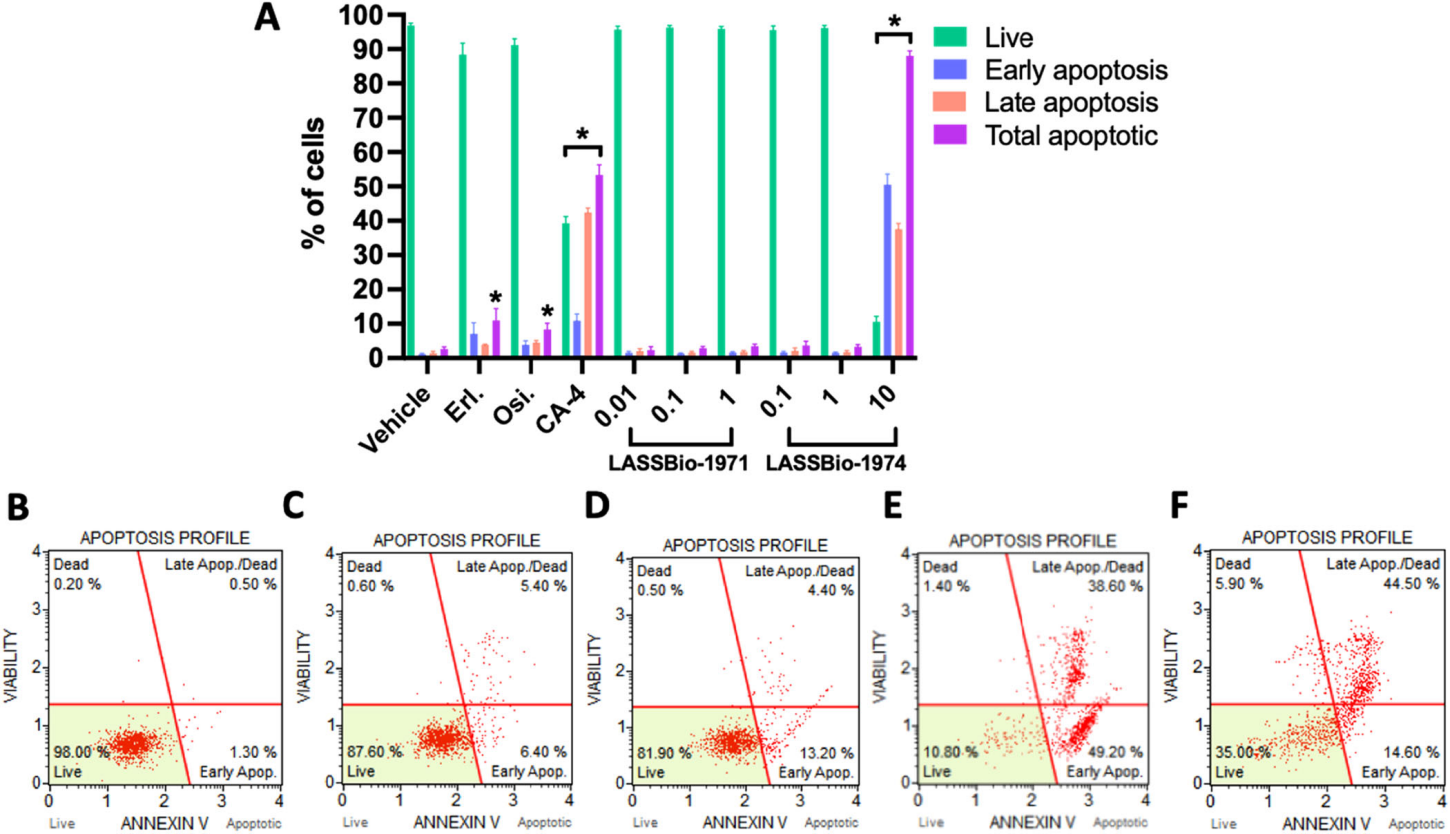
LASSBio‐1971 (**1**) and LASSBio‐1974 (**2**) effect on phosphatidylserine externalization and cell membrane integrity after 72 h incubation in PC‐9_L8585R_. (A) Graph represents mean ± standard error of the mean (*n* ≥ 2); representative dot‐plots from significant *p (< 0.05) value as (B) vehicle (DMSO 1%); (C) Erl.: erlotinib (CC_50_ = 0.1 µM); (D) Osi.: osimertinib (CC_50_ = 0.05 µM); (E) CA‐4: combretastatin A4 (CC_50_ = 0.1 µM); and (F) LASSBio‐1974 (**2**) (CC_50_×10 = 10 µM).

We used flow cytometry to measure apoptosis, a cell death triggering mechanism. Based on the phosphatidylserine externalization and cell membrane fragmentation, LASSBio‐1971 (**1**) could not induce an apoptosis phenotype, exhibiting cell morphology like the untreated control. The EGFRi, erlotinib, and osimertinib demonstrated a modest apoptosis induction at their CC_50_, with a significant increase in the population of cells undergoing apoptosis by approximately 10% (Figure 3).

In contrast, both LASSBio‐1974 (**2**) (10 µM) and combretastatin (CA‐4, 0.1 µM) exhibited high phosphatidylserine externalization and cell membrane permeabilization in nearly 90% and 60% of the NSCLC cell population, respectively. This result corroborates the LASSBio‐1974 (**2**) ability to trigger cell death.

### 2.3 EGFR Inhibition

**Figure 4:**
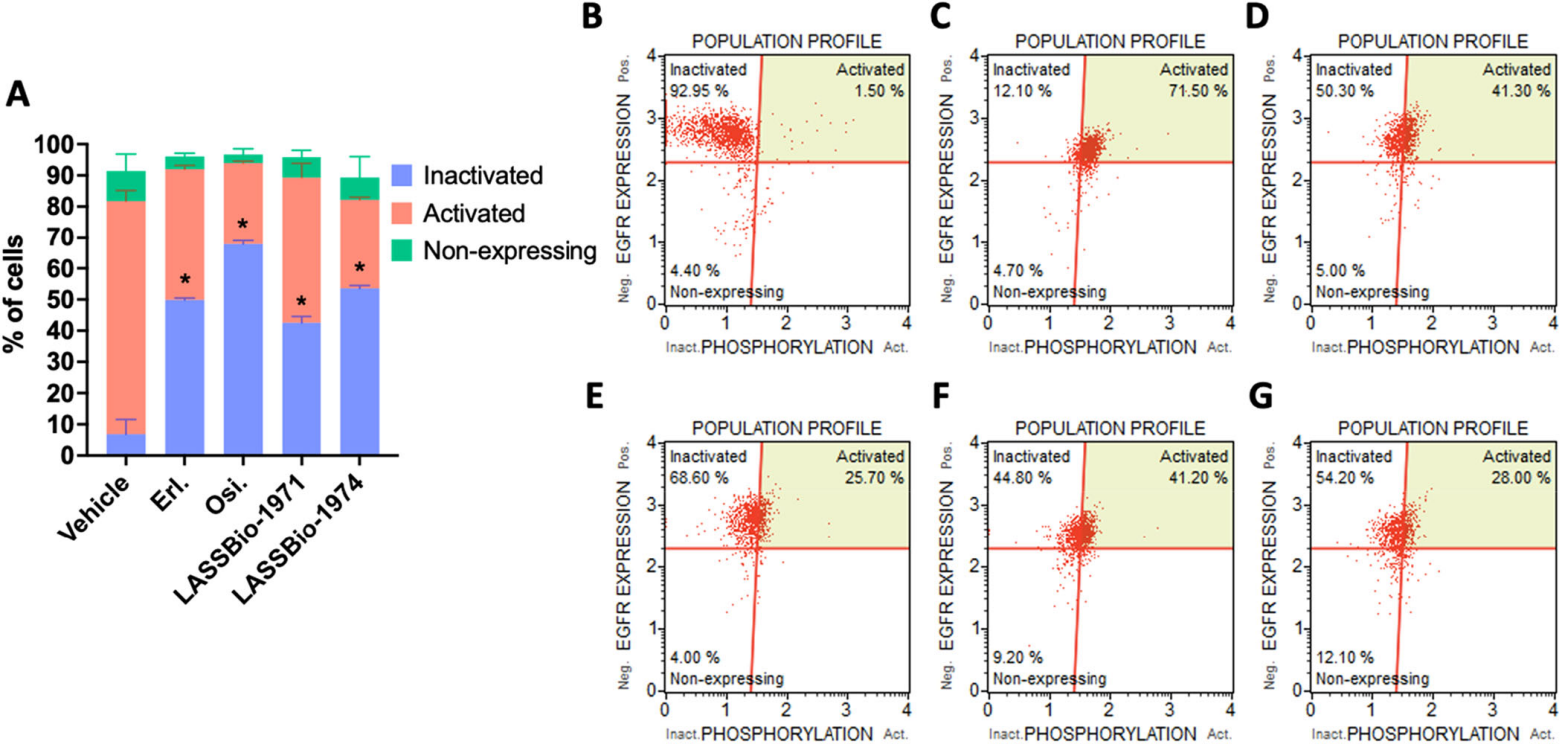
Evaluation of EGFR inhibition in PC‐9_L8585R_ through LASSBio‐1971 (**1**) and LASSBio‐1974 (**2**) activity after 24‐h incubation. (A) Graph represents mean ± standard error of the mean (*n* ≥ 2); representative dot‐plots from significant *p value (< 0.05) as (B) negative control with no antibody anti‐EGFR; (C) vehicle (DMSO 1%); (D) Erl.: erlotinib (CC_50_/0.1 µM); (E) Osi.: osimertinib (CC_50_ = 0.05 µM); (F) LASSBio‐1971 (**1**) (CC_50_ = 0.1 µM); and (G) LASSBio‐1974 (**2**) (CC_50_ = 1 µM).

To attest the LASSBio‐1971 (**1**) and LASSBio‐1974 (**2**) ability to inhibit the EGFR transmembrane protein in a complete cell model in flow cytometry, anti‐EGFR (inactivated) and anti‐phospho‐EGFR (activated) antibodies in flow cytometry were used in this study. It is possible to verify LASSBio‐1971 (**1**) and LASSBio‐1974 (**2**) capability to inhibit phosphorylation and consequently the activation of EGFR in PC‐9_L8585R_ at their respective CC_50_ concentrations when compared to the vehicle group (DMSO 1%) (Figure 4), as previously attested by our research group using in vitro enzymatic model [36] (Figure 2). As expected, erlotinib and osimertinib (EGFRi FDA‐approved drugs) also inhibited phosphorylation and consequently the activation of EGFR (Figure 3).

### 2.4 Cell Cycle Evaluation

To better investigate the mechanism of action of these molecules and assess the inhibition of EGFR given by the arrest of the cell cycle, which is the phase of response to growth factors in the G1 phase, we studied the cell cycle impact of LASSBio‐1971 (**1**) and LASSBio‐1974 (**2**) against PC‐9_L8585R_. LASSBio‐1971 (**1**) caused G0/G1 phase arrest at 1 µM, a consistent characteristic of EGFR inhibition [37], already demonstrated herein, even though it did not trigger apoptotic cell death at the end of 72‐h incubation.

LASSBio‐1974 (**2**) in its CC_50_ (1 µM) induced cycle arresting in the G0/G1 phase, like LASSBio‐1971 (**1**) and the EGFR inhibitor drugs. Unexpectedly, 10 µM of LASSBio‐1974 (**2**) showed cell cycle arrest in G2/M phase, unlike the remarkable EGFR inhibition phenotype. This disparity in cell cycle arrest could be explained by the diversity of action mechanisms that a molecule can perform, as is the case with a molecule with a dual effect [38].

**Figure 5:**
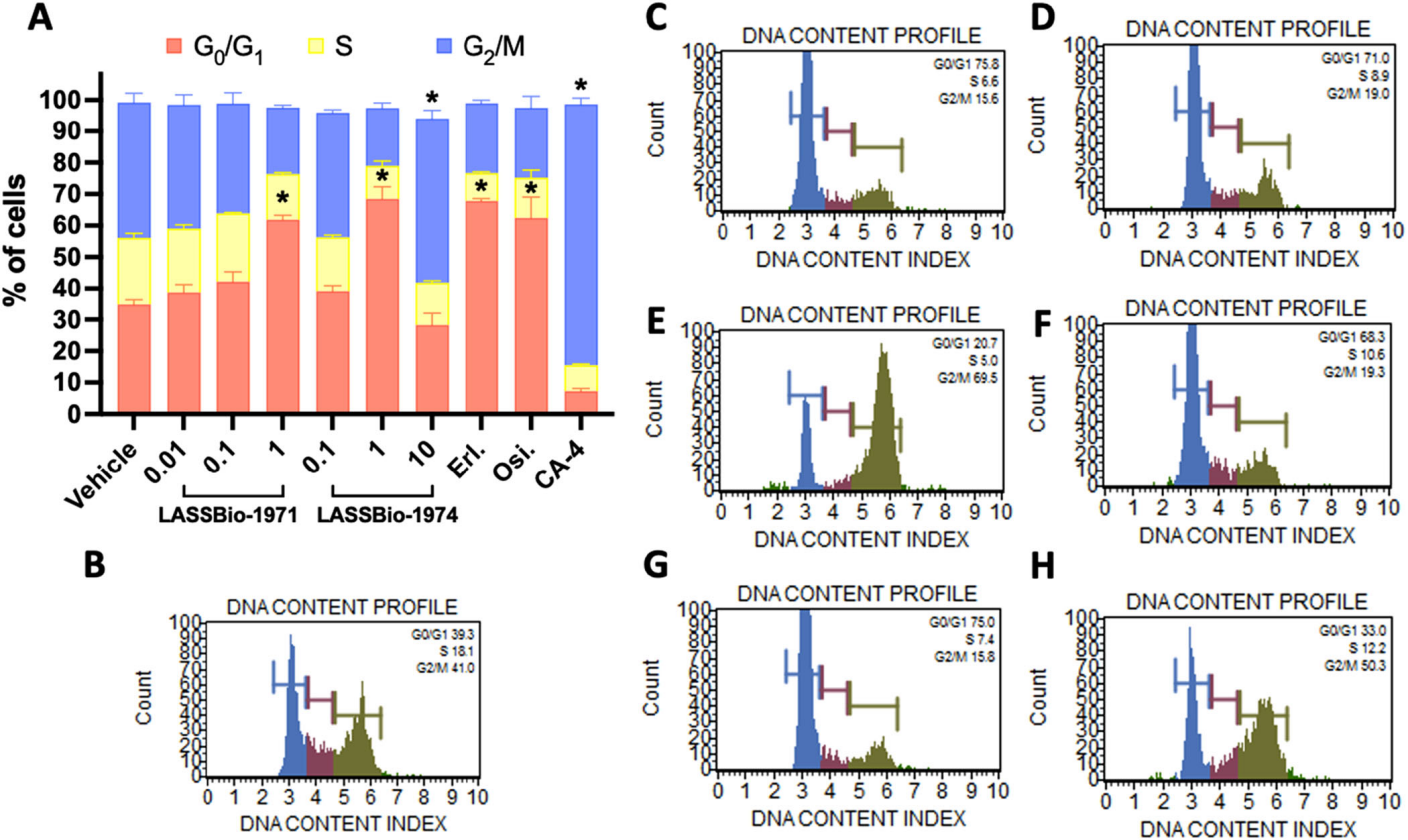
Effect of LASSBio‐1971 (**1**) and LASSBio‐1974 (**2**) on cell cycle progression after 24‐h incubation. *p value was considered significant when < 0.05. (A) Graph represents mean ± standard error of the mean (*n* ≥ 2); representative histograms from significant *p value as (B) vehicle (DMSO 1%); (C) LASSBio‐1971 (**1**) (CC_50_×10 = 1 µM); (D) LASSBio‐1974 (**2**) (CC_50_ = 1 µM); (E) LASSBio‐1974 (**2**) (CC_50_ × 10/10 µM); (F) Erl: erlotinib (CC_50_ = 0.1 µM); (G) Osi: osimertinib (CC_50_ = 0.05 µM); and (H) CA‐4: combretastatin A4 (CC_50_ = 0.1 µM).

As we showed herein (Tables 1 and 2), LASSBio‐1974 (**2**) has demonstrated lack of selectivity against different NSCLC cell lines harboring distinct EGFR mutations, high apoptotic‐inducing potential comparable to CA‐4 (Figure 3), and distinct cell cycle modulation according to the concentration (Figure 5). This evidence led us to speculate that LASSBio‐1974 (**2**) probably has an antimitotic action in addition to EGFR inhibition.

### 2.5 Immunofluorescence Imaging

Microtubules are essential cell morphology filaments, consisting of α/β‐tubulin heterodimers, that are used to build up the cell cytoskeleton structure [39, 40]. Polymerization and depolymerization processes are crucial steps for new filament formation. They happen mainly during the mitosis cell cycle phase, which is necessary for chromosome segregation and cytokinesis. Therefore, anticancer drugs that target microtubules and the mitosis phase generally cause cycle arrest in cells undergoing rapid proliferation, such as tumor cells [41, 42].

Concentrations used in the immunofluorescence imaging experiments were selected based on the CC₅₀ values obtained from the phenotypic assays. This approach allowed us to compare the effects of different kinase inhibitors at biologically relevant concentrations, reflecting their relative potencies in the cellular context, on the flow cytometry assay results. It is suspected that EGFR inhibitors may interact with tubulin during mitosis in the cytoplasm, leading to either stabilization or destabilization of microtubules. Consequently, in this experiment, the following compounds were utilized: combretastatin A4 (CA‐4, 0.03 µM), vincristine (0.03 µM), and paclitaxel (0.03 µM), all as tubulin inhibitors; erlotinib (21 µM) and osimertinib (3 µM) as EGFR inhibitors; and the target compounds LASSBio‐1971 (**1**) (5 µM) and LASSBio‐1974 (**2**) (4 µM).

**Figure 6:**
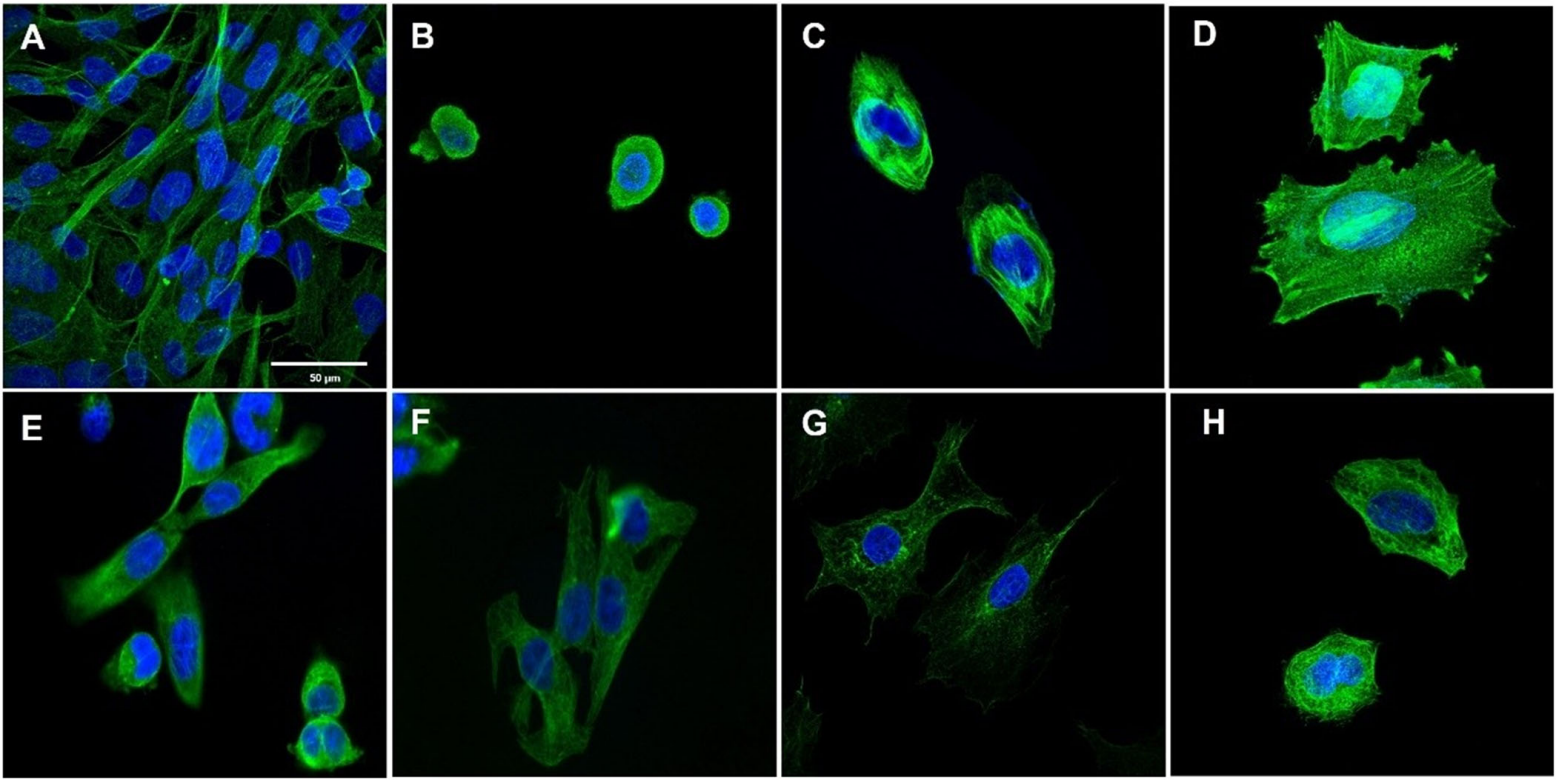
Immunofluorescence of NCI‐H1975 cells. The cells viewed with a 63‐fold objective, were stained with an antibody against β‐tubulin (green) and with DAPI (blue), maintained with or without treatment for 72 h. (A) Negative control (DMSO 0.1%); (B) CA‐4 (0.03 µM); (C) vincristine 4 (0.03 µM); (D) paclitaxel (0.03 µM); (E) erlotinib (21 µM); (F) osimertinib (3 µM); (G) LASSBio‐1971 (**1**) (5 µM); and (H) LASSBio‐1974 (**2**) (4 µM).

LASSBio‐1971 (**1**) and LASSBio‐1974 (**2**) were able to induce morphological changes in cells and to interact with microtubules, resulting in a reduction in cell numbers. Nontreated group of NCI‐H1975 cells exhibited an elongated morphology (Figure 6A). In contrast, cells treated with CA‐4 (Figure 6B) and LASSBio‐1974 (**2**) (Figure 6H) displayed rounded cellular architecture, which is characteristic of microtubule‐destabilizing agents [43]. In groups treated with paclitaxel (Figure 6D) and LASSBio‐1971 (**1**) (Figure 6G), cells exhibit a more spread‐out morphology, suggesting a potential tubulin‐stabilizing effect, where microtubules appear disorganized within the cytoplasm [44]. These findings are particularly noteworthy considering that the replacement of acrylamide by a 4‐dimethylamine‐but‐2‐enamide subunit altered both cellular behavior and the mode of microtubule binding, supporting the hypothesis that these compounds may interact with this target, albeit with reduced potency.

EGFR inhibitors erlotinib and osimertinib (Figure 6E,F) induced a spread‐out morphology, though less pronounced than that observed in the negative control (Figure 6A), especially in the case of erlotinib, where tubulin appears to be concentrated closer to the cell nucleus. The observed morphological changes suggest that tubulin may serve as target for these new kinase inhibitors, especially LASSBio‐1974 (**2**).

### 2.6 Parallel Artificial Membrane Permeability Assay (PAMPA)

The comparative permeability profile of LASSBio‐1971 (**1**) and LASSBio‐1974 (**2**) was investigated using parallel artificial membrane permeability mimicking the gastrointestinal tract (GIT) and the blood–brain barrier (BBB). Regarding PAMPA‐BBB results, LASSBio‐1971 (**1**) was permeable, exhibiting a permeability of 4.16 × 10^−6^ cm/s (Table 3), while LASSBio‐1974 (**2**) showed lower permeation profile (Pe 2.37 × 10^−6^ cm/s), being placed in method indeterminacy range (CNS ±/borderline). The same permeation pattern can be observed at PAMPA‐GIT, once LASSBio‐1971 (**1**) showed high permeation by GIT, with an absorbed fraction (Fa) of 94.62%, while LASSBio‐1974 (**2**) displayed a lower permeability profile with 26.78% (Table 3).

**Table 3:** Parallel artificial membrane permeability assay (PAMPA) for blood–brain barrier (BBB) and gastrointestinal tract (GIT).

Compounds	*Pe*. Exp. BBB (10^−6 ^cm/s)	Classification BBB	*Pe*. Exp. GIT (10^−6 ^cm/s)	Fa (%)	Classification GIT
LASSBio‐1971 (**1**)	4.16 ± 1.84	CNS +	7.69 ± 0.7	94.62	High
LASSBio‐1974 (**2**)	2.37 ± 0.44	CNS ±	0.82 ± 0.47	26.78	Low

*Note:* Results are expressed by mean ± standard deviation (*n* = 3) of experimental permeability.

### 2.7 Metabolic Stability In Vitro

Furthermore, the comparative metabolic stability of LASSBio‐1971 (**1**) and LASSBio‐1974 (**2**) was investigated using RLMs in the presence and absence of a cofactor (NADPH‐generating system).

LASSBio‐1971 (**1**) exhibited low metabolic stability in RLM in the presence of a NADPH‐generating system, suggesting metabolic lability due to the action of oxidative enzymes (CYP450 and/or FMO). It displayed a half‐life (*t*_1/2_) of 53.7 min, and an intrinsic clearance of 3.225 µL/min/mg. LASSBio‐1974 (**2**) was still more unstable, exhibiting a half‐life (*t*_1/2_) of only 5.91 min and an intrinsic clearance of 29.30 µL/min/mg, which suggests its greater lability to oxidative reactions catalyzed by enzymes present in the RLM, probably involving dealkylation steps of the tertiary alkylamine present in its structure. The metabolic stability of LASSBio‐1971 (**1**) and LASSBio‐1974 (**2**) was inverted in RLM studies in the absence of an NADPH‐generating system, a condition in which hydrolysis reactions, catalyzed by carboxylesterase, predominate (Table 4).

**Table 4:** Pharmacokinetic parameters calculated for the in vitro microsomal stability assay to LASSBio‐1971 (**1**) and LASSBio‐1974 (**2**).

Compound	NADPH regenerator system	Metabolism rate (%)	Elimination rate constant (*k*)	*t*_1/2_ (min)	Cl_int_ (µL/min/mg)	Recovery (%)
LASSBio‐1971 (**1**)	Presence	53.93	0.0129	53.72	3.225	90.59
	Absence	21.99	0.0039	177.69	0.975	
LASSBio‐1974 (**2**)	Presence	83.11	0.1172	5.91	29.30	93.17
	Absence	10.78	0.0016	433.12	0.400	

### 2.8 Conjugation With Glutathione

Acrylamide is a molecular fragment that has been widely explored as a warhead for the design of covalent kinase inhibitors, allowing the formation of a covalent bond between its electrophilic carbon and the cysteine residue of the protein kinase binding site, through a Michael‐type addition reaction. This reaction depends on the nucleophilicity of the sulfhydryl present in the cysteine residue and the electrophilicity of carbon 4 of the α,β‐unsaturated carbonyl system. One of the most common models used to investigate the reactivity of various warheads is the glutathione conjugation assay. The ability of a given warhead to bind covalently to glutathione proves the feasibility of forming a covalent bond with cysteine residues but also anticipates the potential levels of off‐target reactivity and toxicity issues, given that C‐S bond formation can occur with different S‐nucleophiles other than those present in the sites of target protein kinases [45, 46, 47, 48].

**Figure 7:**
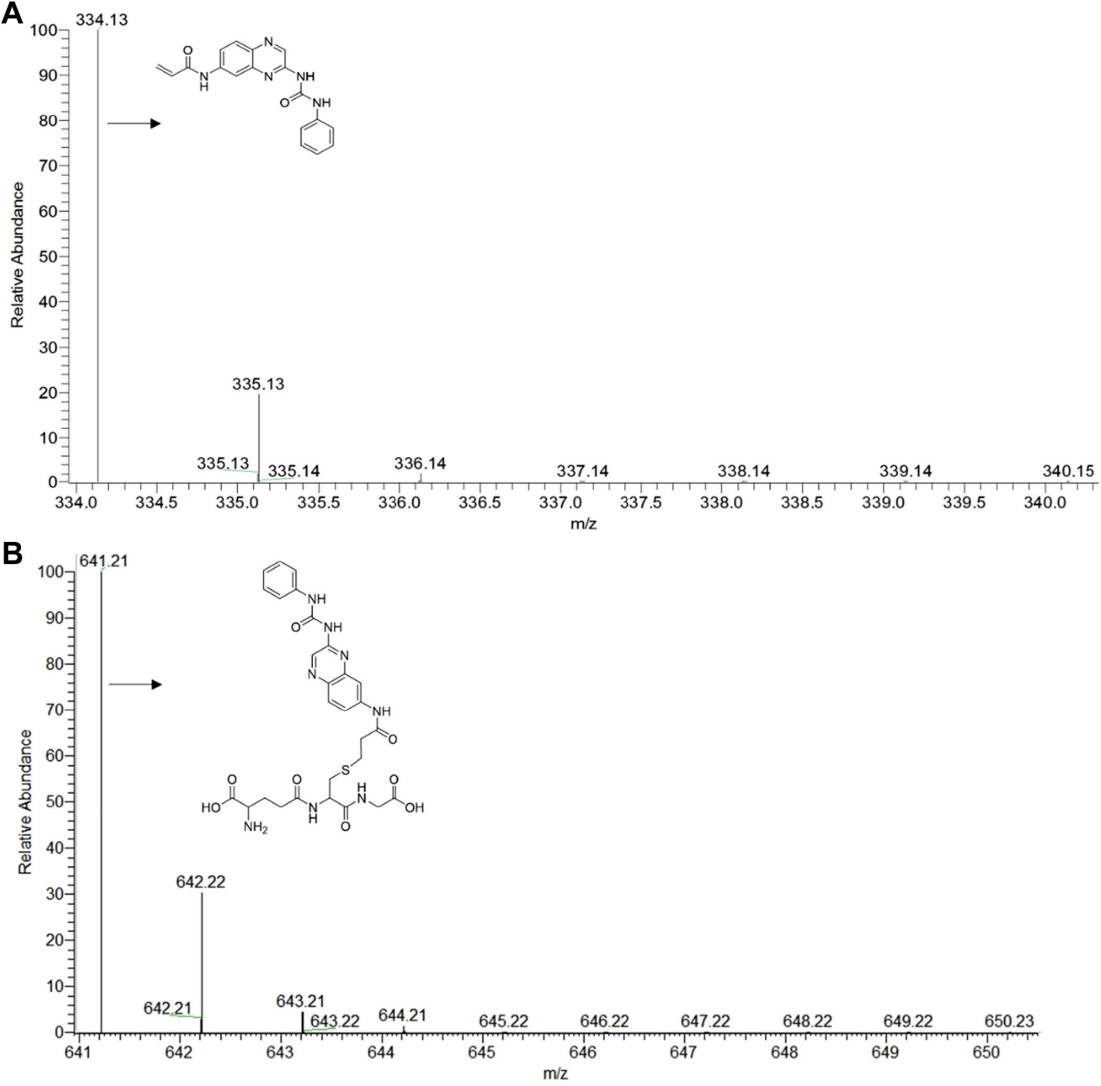
LC‐ESI(−)‐HRMS mass spectra of identification of rat in vitro phase II metabolites of LASSBio‐1971 (**1**); (A) shows LASSBio‐1971 (**1**), the precursor ion of *m/z* 334.13 [M + H]^+^, in time 0 min of incubation and (B) the fragment obtained from the conjugation product of glutathione of *m/z* 641.21 [M + H]^+^.

Therefore, we decided to investigate the ability of LASSBio‐1971 (**1**) to form a covalent adduct with glutathione in the presence of RLMs. As depicted in Figure 7, a covalent adduct between glutathione and LASSBio‐1971 (**1**) was formed and was characterized by LC/MS/MS studies. The presence of a pseudo molecular ion characteristic of conjugation of LASSBio‐1971 (**1**) was confirmed by the comparison of the peak relative to the molecule itself, *m/z* 334.12 [M + H]^+^ (Figure 7A, time 0 min of incubation) and *m/z* 641.21 [M + H]^+^ (Figure 7B, time 60 min of incubation). The mass spectra relative to the standard used and other fragments can be found in the supporting material (Figures S9 and S10).

## 3 Discussions

The most recent EGFRi dacomitinib was approved by the FDA in 2018, also a covalent inhibitor targeting both BTK and EGFR [49, 50]. EGFR is one of the key targets in oncology, and its overactivity drives the progression of NSCLC. Covalent EGFRi were strategically designed with acrylamide Michael acceptors to react with a cysteine residue (Cys797) in EGFR [49]. Nonetheless, these inhibitors could display poor selectivity due to their high reactivity, associated with the presence of different types of warheads. LASSBio‐1971 (**1**) and LASSBio‐1974 (**2**) were previously reported as covalent EGFR inhibitors, presenting a new molecular framework, and exhibiting activity in the nanomolar range on the wild‐type and mutant EGFR enzyme L858R. Only LASSBio‐1974 (**2**) proved potent in inhibiting the doubly mutated EGFR (EGFR_L858R/T790M_). LASSBio‐1971 (**1**) displayed IC_50_ values of 25 ± 4 nM for EGFR_wt_, 18 ± 3 nM for EGFR_L858R_, and 1682 ± 1506 nM for EGFR_L858R/T790M_, compared to LASSBio‐1974 (**2**), which showed IC_50_ values of 101 ± 12, 32 ± 14, and 132 ± 49 nM, respectively [36]. Therefore, considering the potency of these important hits on EGFR inhibition, studying their cytotoxic potential on overexpressing‐EGFR NSCLC cell lines NCI‐H292 (EGFR_wt_), PC‐9 (EGFR_L858R_), and NCI‐H1975 (EGFR_L858R/T790M_) would provide information on the convergence of enzymatic and phenotypic assays.

The results obtained (Tables 1, 2, and S1), and compared with erlotinib and osimertinib, confirm the cytotoxic effect measured by two different methods, and are compatible with the enzyme potency results. LASSBio‐1971 (**1**), which has the highest potency on EGFR_wt_ and EGFR_L858R_, was the one with the lowest CC_50_ values on NCI‐H292 and PC9 (CC_50_ = 1.51 and 0.13 µM, respectively). In general, both hits were equipotent on the three NSCLC cell lines studied, but less potent than the standard drugs. The ability of these hits to exert cytotoxic activity by modulating the EGFR pathway was investigated and confirmed (Figure 3). Flow cytometry assays revealed a distinct concentration‐dependent profile between LASSBio‐1971 (**1**) and LASSBio‐1974 (**2**), suggesting the latter's ability to act as an antimitotic agent (Figure 4), which was reinforced by immunofluorescence analysis (Figure 5). Therefore, the data propose that at the highest concentration of 10 µM, LASSBio‐1974 (**2**) may exert dual action on NCI‐H1975 cells, inhibiting EGFR and modulating microtubule formation.

A range of other new molecules has shown dual EGFR/tubulin inhibition strategies to treat EGFR‐aberrant cancer [32, 51, 52, 53]. Recently, promising other molecules were designed and reported with potential for dual inhibition of EGFR and microtubules with anticancer activity [54, 55, 56, 57], including LASSBio‐2070 developed by our group [58].

Briefly, our results demonstrated antiproliferative activity through EGFR inhibition and cell cycle arrest in G0/G1 against NSCLC cell lines to LASSBio‐1971 (**1**). On the other hand, LASSBio‐1974 (**2**) showed cytotoxic activity through induction of apoptosis and cell cycle arrest at G0/G1 and G2/M phases (concentration‐dependent) as a possible dual mechanism of action. Thus, LASSBio‐1974 (**2**) may appear as a dual‐target inhibitor for NSCLC treatment, and possibly other cancer types with EGFR disturbances. Even though our data shows this potential, further investigations are needed to understand the specific target of LASSBio‐1974 (**2**) other than EGFR and how this compound arrests cell cycle in G2/M phase, as well as its consequences in cell signaling, which may include western blot analysis of phospho‐Histone H3 along with β‐tubulin molecular docking studies, and competitive binding assays for precise mechanistic characterization.

Characterizing the in vitro pharmacokinetic profile is an important step in early drug discovery [59]. For this reason, once the pharmacodynamic profile of action of LASSBio‐1971 (**1**) and LASSBio‐1974 (**2**) was confirmed, their permeability, half‐life, and intrinsic clearance were studied. We demonstrated that LASSBio‐1971 (**1**) was highly permeable by PAMPA‐GIT, unlike LASSBio‐1974 (**2**). LASSBio‐1971 (**1**) also demonstrated greater permeability on PAMPA‐BBB. This data, together with the description that EGFR induces proliferation and/or has a trophic effect on multiple cell types [20, 23], including glioblastomas, where the EGFR gene can be amplified and overexpressed [60], may indicate the possibility of repositioning LASSBio‐1971 (**1**) for studies in glioblastoma models. In fact, recently, our group has demonstrated the cytotoxic activity of LASSBio‐1971 (**1**) alone and in association with gedatolisib in human glioblastoma cell lines, as well as its BBB‐permeability on HBMEC monolayer assay [61].

The half‐life and hepatic clearance of LASSBio‐1971 (**1**) and LASSBio‐1974 (**2**) in RLM showed that despite the presence of two potentially hydrolysable groups (amide and urea), clearance occurs preferentially by oxidative metabolism (Table 3) and that LASSBio‐1971 has the greatest metabolic stability and therefore the longest half‐life and lowest clearance.

Finally, given the presence of a Michael acceptor site introduced in both hits to enable covalent inhibition of EGFR, we investigated the most promising compound, LASSBio‐1971 (**1**), for its ability to form covalent adducts with glutathione in the presence of RLM. The formation of this adduct was confirmed by LC/MS/MS, suggesting that other cysteine‐based nucleophiles could also form covalent bonds with LASSBio‐1971 (**1**), which raises potential safety concerns.

Structurally, both compounds were designed with an electrophilic warhead positioned to react with the Cys797 residue in the ATP‐binding pocket, a well‐established target for covalent EGFR inhibitors. Initially, noncovalent interactions stabilize the ligand within the binding pocket, properly orienting the electrophilic warhead toward Cys797. The subsequent covalent bond formation represents a crucial step that converts a reversible interaction into an irreversible inhibition event, significantly enhancing potency, selectivity, and duration of action.

Consistently, the sustained inhibitory activity observed in our cellular assays—when compared with reversible EGFR inhibitors tested under the same conditions—supports the occurrence of irreversible target engagement. Taken together, these data strongly suggest that covalent bond formation is a major contributor to the inhibitory effect of both molecules. Indeed, this covalent mechanism ensures prolonged target engagement and sustained inhibition even after free drug clearance, aligning these compounds mechanistically with modern covalent EGFR inhibitors, such as osimertinib, which were developed to overcome resistance associated with earlier generations of EGFR inhibitors. Therefore, while both molecules benefit from favorable reversible interactions that drive affinity, it is the irreversible modification of Cys797 that primarily determines their overall pharmacological effect.

## 4 Conclusions

Taken together, our data pointed to LASSBio‐1971 (**1**) as an EGFR inhibitor with good cytotoxic potency on human NSCLC lines containing different expressions of the wild‐type and mutant forms of the protein. It also shows good permeation capacity on membranes that mimic the GIT and the BBB, as well as good metabolic stability. However, the identification of a covalent adduct with glutathione raises a yellow light regarding safety issues for the possible continued use of this compound. Its structural analog, LASSBio‐1974 (**2**), exhibited similar cytotoxic potency but lower permeation properties and metabolic stability than LASSBio‐1971 (**1**). Further studies should be conducted with LASSBio‐1974 (**2**) to prove and understand its microtubule action.

Future studies are planned to further evaluate the selectivity of both compounds, including broader cysteine‐reactivity profiling and additional kinase panels, to better characterize potential off‐target interactions. Concerning LASSBio‐1974 (**2**), it is important to provide additional evidence for its antimitotic activity, performing complementary assays, such as tubulin polymerization and binding studies, to directly confirm its effect on microtubules and further elucidate its mechanism of action.

## 5 Experimental

### 5.1 Cell Culture

Overexpressing‐EGFR NSCLC cell lines NCI‐H292 (EGFR_wt_), PC‐9 (EGFR_L858R_), and NCI‐H1975 (EGFR_L858R/T790M_) were all purchased from the Cell Bank of Rio de Janeiro (BCRJ) (Rio de Janeiro, Brazil). Cells were grown in RPMI‐1640 medium supplemented with 10% fetal bovine serum following BCRJ's recommendations. All cell lines were harvested at 37°C and 5% CO_2_ atmosphere humid incubator.

### 5.2 Cell Viability by MTT Assay

Cells were plated at 1 × 10^4^ to 3 × 10^4^ cells/well and seeded in a 96‐well plate. After 16–24 h of cell acclimatization, compounds were added at 0.003–100 µM concentrations (1% DMSO vehicle). After 72 h, treatment was interrupted to measure cell viability using MTT (3‐(4,5‐dimethylthiazol‐2‐yl)‐2,5‐diphenyltetrazolium bromide) (50 µg/mL) [62]. Formazan formed by viable cells was solubilized after 4 h incubation with SDS/HCl solution (0.1 g/mL) and read at 570 nm in spectrophotometer. CC_50_ values were calculated with nonlinear regression of data in quadruplicate of at least three independent experiments (*n* ≥ 3) using GraphPad Prism (version 8.0).

### 5.3 Cell Viability by Sulforhodamine (SRB) Assay

Cells were plated at 1 × 10^4^ to 3 × 10^4^ cells/well and seeded in a 96‐well plate. After 16–24 h of cell acclimatization, compounds were added at 0.003–100 µM concentrations (1% DMSO as vehicle). The experimental procedure was handled as described by Vichai and Kirtikara [63]. After incubation, cells were fixed with trichloroacetic acid (TCA) (10% m/v) for 1 h at 4°C. SRB (0.004% w/v in acetic acid 1%) was added for 30 min and washed with acetic acid 1%. Dried samples were solubilized with Tris‐base solution (pH 10.5) and read at 560 nm UV spectrophotometer. The CC_50_ values were determined by nonlinear regression analysis, based on quadruplicate data obtained from at least three independent experimental replicates (*n* ≥ 3), using GraphPad Prism software (version 8.0).

### 5.4 Flow Cytometry

PC‐9 (EGFR_L858R_) cell line was seeded at 1 × 10^5^ cells/well in a 24‐well plate. Then, each respective CC_50_/10, CC_50_, and/or 10× CC_50_ values of LASSBio‐1971 (**1**) and LASSBio‐1974 (**2**), EGFR inhibitors such as osimertinib (WZ4002, Sigma‐Aldrich) and erlotinib (Sigma‐Aldrich), and the non‐EGFRi combretastatin A‐4 (CA‐4) (Sigma‐Aldrich) were added at their CC_50_ concentration. All flow cytometry assays were performed using Guava Muse Cell Analyzer (Cytek Biosciences) following each kit user's guide provided by the manufacturer. Muse Cell Cycle Kit (catalog no. MCH100106) was chosen to evaluate cell cycle arresting. Apoptosis features were evaluated using Muse Annexin V & Dead Cell Kit (catalog no. MCH100105), as well as inactivation of EGFR using Muse EGFR‐RTK Activation Dual Detection Kit (catalog no. MCH200102). Statistical analysis was performed using one‐way ANOVA on data collected in duplicate from at least two independent experiments (*n* ≥ 2), employing GraphPad Prism software (version 8.0).

### 5.5 Immunofluorescence and Digital Image Acquisition

NCI‐H1975 (EGFR_L858R/T790M_) cell line was chosen for a better visualization of the effects of the compounds on microtubules. The cells were cultured and incubated for 24 h with 0.1% DMSO (negative control group), 0.03 µM combretastatin A4, 0.03 µM paclitaxel, 0.03 µM vincristine, 21 µM erlotinib, 3 µM osimertinib, 5 µM LASSBio‐1971 (**1**), and 4 µM LASSBio‐1974 (**2**). Every concentration is 10 times less than CC_50_ in 24 h by MTT assay, except for tubulin inhibitors. After the incubation period, they were fixed with 4% paraformaldehyde and permeabilized with 0.5% Triton‐X 100 in PBS and after subsequent washes, incubated with primary antibody, and later with Alexa Fluor 488, secondary antibody conjugated both for 1 h at 37°C, and the nucleus was labeled with DAPI (0.1 mg/mL in 0.9% NaCl) [64]. Cells were photographed on a Leica TCS SPE confocal microscope (Leica Microsystems, Germany) using oil immersion 63× and acquired by LAS X Programs, with the effects of the compounds analyzed from changes in cell morphology by the NIH ImageJ public domain program (developed at the National Institutes of Health and available at http://imagej.nih.gov/ij/).

### 5.6 PAMPA

For BBB and GIT, PAMPA was used a donor plate where the compounds (tests or controls) were diluted in buffered medium, which is characterized by the presence of a synthetic membrane of PVDF (polyvinylidene fluoride) impregnated with a lipid solution, forming a barrier through which the compounds migrate through a diffusion process to the lower plate called the receptor [65, 66, 67]. The lipid mixture that impregnates the filter has a different constitution for the permeability tests for the BBB (brain lipid from pig extract in *n*‐dodecane) and the GIT (L‐α soy phosphatidylcholine in *n*‐dodecane). The optical density values obtained in the reading at each selected wavelength, for each of the compounds, were analyzed in comparison with the values of several controls within a calibration curve (≥ 7 drugs). In the case of the BBB, these values were used to elaborate an equation and determine the permeability coefficient (Pe), while GIT used absorbed fraction (Fa%). The permeability result for PAMPA‐GIT classifies the compounds according to the percentage of absorbed fraction (Fa%), as: high intestinal permeability (70%–100%), medium permeability (30%–69%), or low permeability (0%–29%), and the samples were diluted from stock solution of 10 mM [43]. The PAMPA‐BBB model classifies the compounds only as: permeable (central nervous system, CNS+) or non‐permeable (CNS−), and the assays were made from stock solution of 1 mg for each compound [67, 68, 69].

### 5.7 Conjugation With Glutathione

Incubation with pooled RLMs (1 mg/mL protein) was conducted in 50 mM potassium phosphate buffer (pH 7.4) at 37°C in an incubator for 60 min. LASSBio‐1971 (**1**) and P‐chlorophenyl isocyanate (10 μM) and 2 mM GSH were added to the buffer. The incubation was initiated by NADPH‐generating system consisting of 40 mM NADP^+^, 350 mM glucose‐6‐phosphate, 5 units/mL glucose‐6‐phosphate dehydrogenase, and 130 mM MgCl_2_. The incubation was terminated at 60 min by the addition of 1 mL of ACN (with 6% of formic acid, with a total volume of 1.25 mL). The quenched sample was then centrifuged at 10,500 × *g* for 15 min, and 1 mL of the supernatant was collected to be injected into the Q‐Exactive Plus ESI MS System (Thermo Scientific, USA) [70, 71].

### 5.8 Microsomal Metabolic Stability

The metabolic stability of compounds LASSBio‐1971 (**1**) and LASSBio‐1974 (**2**) (10 μM) was incubated in 1 mg/mL of rat microsomes in the presence and absence of the cofactor (i.e., NADPH‐generating system) at the same concentrations as in the previous section. After mixing and incubating at 37°C in a shaking water bath for 1 h, 500 µL cold acetonitrile containing the internal standard was added to terminate the reaction at different time intervals (0, 15, 30, 45, and 60 min). Samples were mixed and centrifugated at 10,500 × *g* for 15 min at 4°C, the supernatant was filtered and injected into the HPLC system using a mixture of methanol: water acidified (0.1% formic acid, pH 3.0) (50:50, v/v) as the mobile phase at a flow rate of 1.0 mL/min Pharmacokinetic parameters were calculated as microsomal half‐life (*t*_1/2_) and intrinsic clearance (Cl_int_) [72].

## Conflicts of Interest

The authors declare no conflicts of interest.

## Supporting information

Supp information.
**Table S1:** LASSBio‐1971 (**1**) and LASSBio‐1974 (**2**) cytotoxic activity against NSCLC cell line in 48h period assessed by MTT assay. Results are expressed in micromolar (µM) and confidence interval (95%), (n ≥ 3).
**Table S2:** LASSBio‐1971 (1) and LASSBio‐1974 (**2**) cytotoxic activity against NSCLC cell line in 24h period assessed by MTT assay. Results are expressed in micromolar (µM) and confidence interval (95%), (n ≥ 3).
**Table S3:** Permeability coefficient of standard drugs, used as control and of target compounds by the PAMPA‐BBB technique.
**Table S4:** Permeability coefficient of standard drugs, used as control and of target compounds by the PAMPA‐GIT technique.
**Figure S1:** Cytotoxicity curves of compounds LASSBio‐1971 (**1**) and LASSBio‐1974 (**2**) against NSCLC cell lines in a 72h period assessed by (**A**) MTT assay and (**B**) SRB assay.
**Figure S2:** Cytotoxicity curves of compounds LASSBio‐1971 (**1**) and LASSBio‐1974 (**2**) against NSCLC cell lines assessed by MTT assay in a (**A**) 48h and (**B**) 24h period.
**Figure S3:** Linear correlation between experimental and literature permeability (Pe) values. Data represent the mean of triplicates in two different analyses (n **=** 2).
**Figure S4:** Linear correlation between experimental and literature permeability (Pe) values. Data represent the mean of triplicates in two different analyses (n = 2).
**Figure S5:** A) Liver microsomal stability profile of LASSBio‐1971 (1); B) First order rate constant (*k*) for elimination. Experiment carried out in the presence of a NADPH generating system.
**Figure S6:** A) Liver microsomal stability profile of LASSBio‐1971 (**1**); B) First order rate constant (*k*) for elimination. Experiment carried out in the absence of a NADPH generating system.
**Figure S7:** A) Liver microsomal stability profile of LASSBio‐1974 (**2**); B) First order rate constant (*k*) for elimination. Experiment carried out in the presence of a NADPH generating system.
**Figure S8:** A) Liver microsomal stability profile of LASSBio‐1974 (**2**); B) First order rate constant (*k*) for elimination. Experiment carried out in the absence of a NADPH generating system.
**Figure S9:** LC‐ESI(+)‐HRMS mass spectra of identification of rat in vitro phase II metabolites of *P*‐chlorophenyl isocyanate, used as a standard for method validation; **A**) shows P‐chlorophenyl isocyanate, the precursor ion of m/z 154.01 [M + H]^+^; and **B**) the fragment obtained from the conjugation product of glutathione of m/z 475.10 [M + H]^+^. Figure A shows the compound in time 0 minute of incubation.
**Figure S10:** LC‐ESI(‐)‐HRMS mass spectra of identification of rat in vitro phase II metabolites of LASSBio‐1971 (**1**); **A**) the fragment obtained from the conjugation product of glutathione of m/z 512.17 [M + H]^+^; and **B**) the fragment obtained from the conjugation product of glutathione of m/z 583.21 [M + H]^+^.
