# Supplementary material for: Investigation on LASSBio‐1971 and LASSBio‐1974 Cellular Cytotoxic Mechanism and Their Comparative DMPK Profile
Source: Arch Pharm (Weinheim). 2025 Nov 21;358(11):e70151. doi: 10.1002/ardp.70151 (PMC12637173; doi:10.1002/ardp.70151)

**Supplementary material**

**INVESTIGATION ON LASSBio-1971 AND LASSBio-1974 CELLULAR CYTOTOXIC MECHANISM AND THEIR COMPARATIVE DMPK PROFILE**

Manoel Oliveira de Moraes Junior^1,2^, Gisele Barbosa^1,2^, Caroline Marques Xavier da Costa^1,2^, Raysa Magali Pillpe-Meza^1,2^, Wesley Leandro de Gouveia^1,2^, Daniel Nascimento do Amaral^1,2^, Luis Gabriel Valdivieso Gelves^1^, Stefan Laufer^1,3^, Lídia Moreira Lima^1,2*^

^1^ National Institute of Science and Technology of Pharmaceuticals and Medications (INCT-INOFAR; http://www.inct-inofar.ccs.ufrj.br/). Federal University of Rio de Janeiro, Laboratory of Evaluation and Synthesis of Bioactive Substances (LASSBio®, http://www.lassbio.icb.ufrj.br), CCS, University City, P.O. Box 68024, ZIP: 21941-971, Rio de Janeiro, Brazil.

^2^ Postgraduate Program in Pharmacology and Medicinal Chemistry, Institute of Biomedical Sciences, Federal University of Rio de Janeiro, Rio de Janeiro, Brazil.

^3^ Department of Pharmaceutical Chemistry, IPS, Eberhard Karls Universität Tübingen, Tübingen, Germany.

*Corresponding author:

Lídia Moreira Lima, PhD

Full Professor

Federal University of Rio de Janeiro, Centro de Ciências da Saúde, Bloco B, sala 14, P.O. Box 68024, Rio de Janeiro, RJ, Brasil, ZIP Code: 21941-971.

Phone: +55 21 39386503

Email: [lmlima23@gmail.com](mailto:lmlima23@gmail.com)

**Table and figure legend:**

Table S1: LASSBio-1971 (**1**) and LASSBio-1974 (**2**) cytotoxic activity against NSCLC cell line in 48h period assessed by MTT assay. Results are expressed in micromolar (µM) and confidence interval (95%), (n ≥ 3).

Table S2: LASSBio-1971 (**1**) and LASSBio-1974 (**2**) cytotoxic activity against NSCLC cell line in 24h period assessed by MTT assay. Results are expressed in micromolar (µM) and confidence interval (95%), (n ≥ 3).

Table S3: Permeability coefficient of standard drugs, used as control and of target compounds by the PAMPA-BBB technique.

Table S4: Permeability coefficient of standard drugs, used as control and of target compounds by the PAMPA-GIT technique.

Figure S1: Cytotoxicity curves of compounds LASSBio-1971 (**1**) and LASSBio-1974 (**2**) against NSCLC cell lines in a 72h period assessed by (**A)** MTT assay and (**B)** SRB assay.

Figure S2: Cytotoxicity curves of compounds LASSBio-1971 (**1**) and LASSBio-1974 (**2**) against NSCLC cell lines assessed by MTT assay in a (**A)** 48h and (**B)** 24h period.

Figure S3: Linear correlation between experimental and literature permeability (Pe) values. Data represent the mean of triplicates in two different analyses (n=2).

Figure S4: Linear correlation between experimental and literature permeability (Pe) values. Data represent the mean of triplicates in two different analyses (n=2).

Figure S5: A) Liver microsomal stability profile of LASSBio-1971 (**1**); B) First order rate constant (*k*) for elimination. Experiment carried out in the presence of a NADPH generating system.

Figure S6: A) Liver microsomal stability profile of LASSBio-1971 (**1**); B) First order rate constant (*k*) for elimination. Experiment carried out in the absence of a NADPH generating system.

Figure S7: A) Liver microsomal stability profile of LASSBio-1974 (**2**); B) First order rate constant (*k*) for elimination. Experiment carried out in the presence of a NADPH generating system.

Figure S8: A) Liver microsomal stability profile of LASSBio-1974 (**2**); B) First order rate constant (*k*) for elimination. Experiment carried out in the absence of a NADPH generating system.

Figure S9: LC-ESI(+)-HRMS mass spectra of identification of rat in vitro phase II metabolites of *P*-chlorophenyl isocyanate, used as a standard for method validation; **A**) shows *P*-chlorophenyl isocyanate, the precursor ion of m/z 154.01 [M + H]^+^; and **B**) the fragment obtained from the conjugation product of glutathione of m/z 475.10 [M + H]^+^. Figure A shows the compound in time 0 minute of incubation.

Figure S10: LC-ESI(-)-HRMS mass spectra of identification of rat in vitro phase II metabolites of LASSBio-1971 (**1**); **A**) the fragment obtained from the conjugation product of glutathione of m/z 512.17 [M + H]^+^; and **B**) the fragment obtained from the conjugation product of glutathione of m/z 583.21 [M + H]^+^.

**Contents**

Biological section

Pharmacokinetics section

**Biological section**

**Table S1:** LASSBio-1971 (**1**) and LASSBio-1974 (**2**) cytotoxic activity against NSCLC cell line in 48-hour period assessed by MTT assay. Results are expressed in micromolar (µM) and confidence interval (95%), (n ≥ 3).

| **Compound** | **Cell lines** | | |
| --- | --- | --- | --- |
|  | **NCI-H292 (EGFR*^w.t^*^.^*)*** | **PC-9 (EGFR^L858R^)** | **NCI-H1975 (EGFR^L858R/T790M^)** |
| Erlotinib | 47.31 | 1.31 | >100 |
|  | (40.48 – 55.29) | (0.2 – 8.4) |  |
| Osimertinib | 6.81 | 0.17 | 0.38 |
|  | (5.76 – 8.05) | (0.09 – 0.3) | (0.21 – 0.68) |
| LASSBio-1971 (**1**) | 8.85 | 0.14 | 6.45 |
|  | (6.51 – 12.04) | (0.05 – 0.39) | (4.71 – 8.84) |
| LASSBio-1974 (**2**) | 7.25 | 1.22 | 6.58 |
|  | (5.73 – 9.15) | (0.57 – 2.6) | (4.13 – 10.48) |

**Table S2:** LASSBio-1971 (**1**) and LASSBio-1974 (**2**) cytotoxic activity against NSCLC cell line in 24-hour period assessed by MTT assay. Results are expressed in micromolar (µM) and confidence interval (95%), (n ≥ 3).

| **Compounds** | **Cell lines** | | |
| --- | --- | --- | --- |
|  | **NCI-H292 (EGFR^w.t.^*)*** | **PC-9 (EGFR^L858R^)** | **NCI-H1975 (EGFR^L858R/T790M^)** |
| Erlotinib | >50 | >100 | >100 |
| Osimertinib | ND* | 8.32 | >30 |
|  |  | (5.18 – 13.78) |  |
| LASSBio-1971 (**1**) | 18.35 | 6.42 | 51.34 |
|  | (9.71 – 34.7) | (4.06 – 10.14) | (32.22 – 81.81) |
| LASSBio-1974 (**2**) | 16.39 | 9.74 | 37.97 |
|  | (12.28 – 21.86) | (6.76 – 14.18) | (30.05 – 47.97) |

*ND = not determined

**Figure S1**: Cytotoxicity curves of compounds LASSBio-1971 (**1**) and LASSBio-1974 (**2**) against NSCLC cell lines in a 72-hour period assessed by (**A)** MTT assay and (**B)** SRB assay. Results are expressed as mean ± standard error of the mean (n ≥ 3).


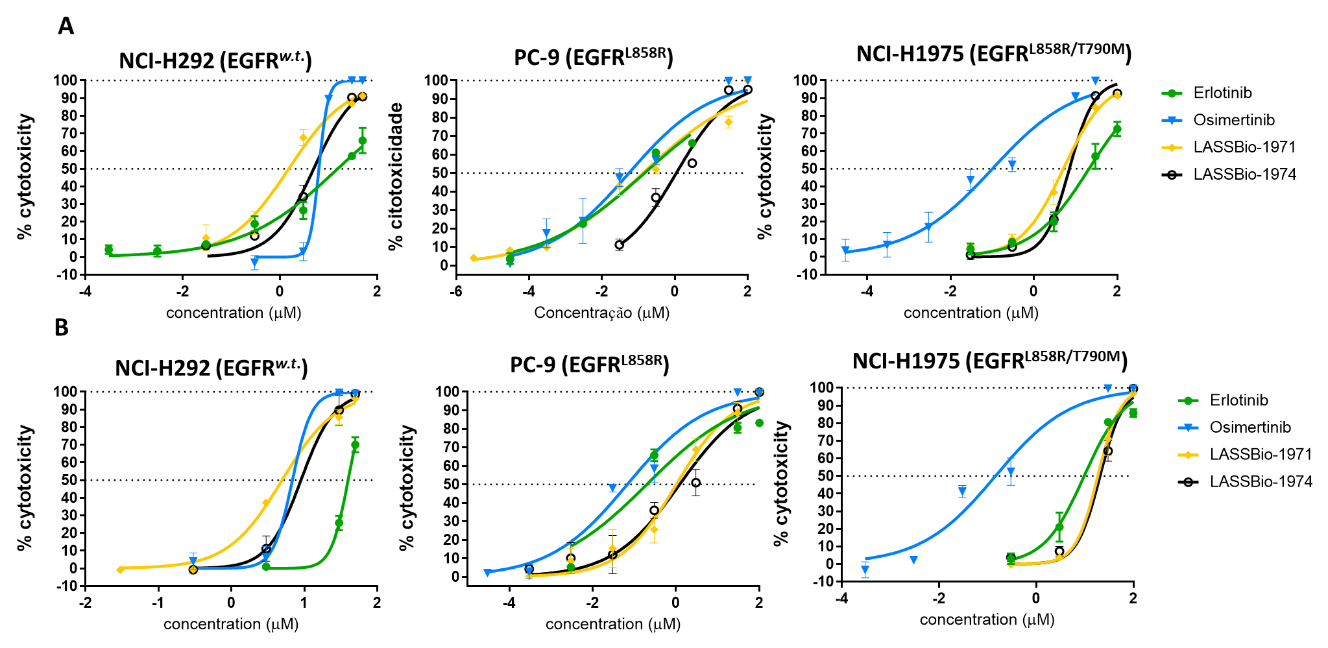


**Figure S2**: Cytotoxicity curves of compounds LASSBio-1971 (**1**) and LASSBio-1974 (**2**) against NSCLC cell lines assessed by MTT assay in a (**A)** 48-hour and (**B)** 24-hour period. Results are expressed as mean ± standard error of the mean (n ≥ 3).


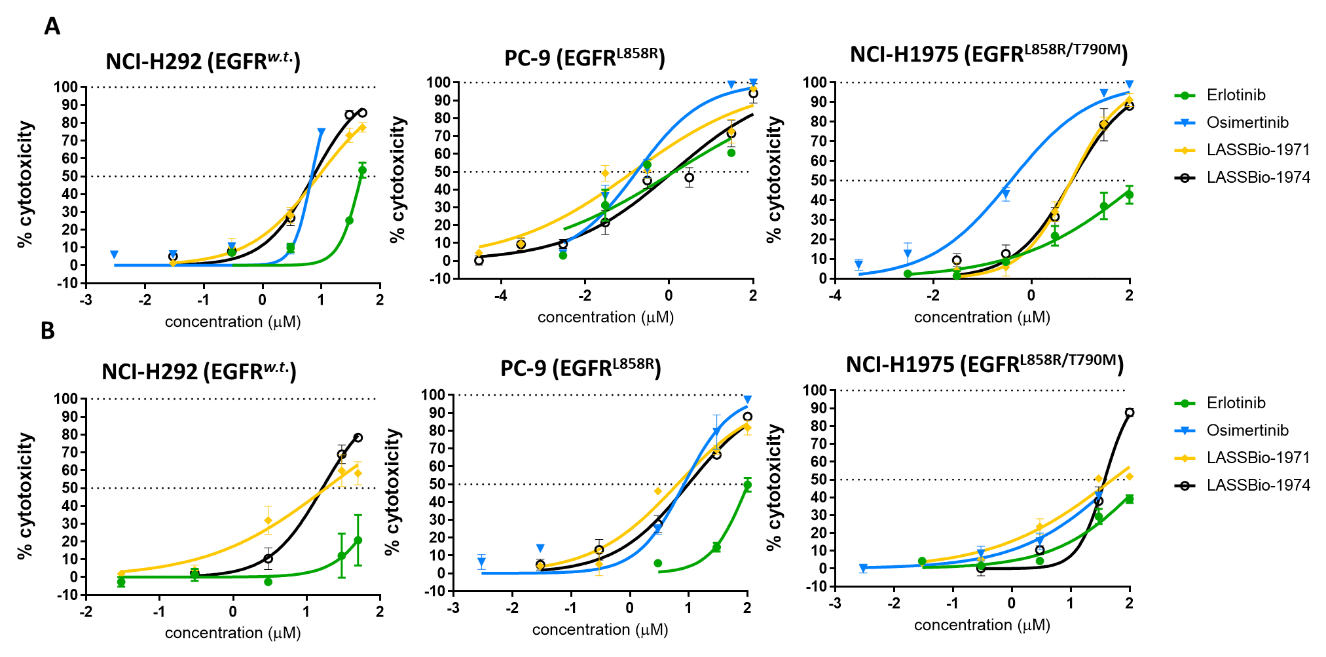


**Pharmacokinetics section**

**Table S3.** Permeability coefficient of standard drugs, used as control and of target compounds by the PAMPA-BBB technique.

| **Compounds** | ***Pe****  **literature**  **(10^-6^ cm s^-1^)** | ***Pe****  **experimental**  **(10^-6^ cm s^-1^)** | **Classification** |
| --- | --- | --- | --- |
| **Atenolol** | **0.8** | 0.59 | CNS - |
| **Caffeine** | **1.3** | 1.28 | CNS - |
| **Enoxacin** | **0.9** | 0.56 | CNS + |
| **Hidrocortisone** | **1.9** | 1.72 | CNS - |
| **Ofloxacin** | **0.8** | 0.54 | CNS - |
| **Testosterone** | **17** | 10.55 | CNS + |
| **Verapamil** | **16** | 8.18 | CNS + |

Results are expressed by mean ± standard deviation (n = 2) of experimental permeability (Pe. Exp, 10^-6^ cm/s). **Pe.*: Permeability; CNS.: central nervous system.

**Figure S3.** Linear correlation between experimental and literature permeability (Pe) values. Data represent the mean of triplicates in two independent analyses (n = 2).


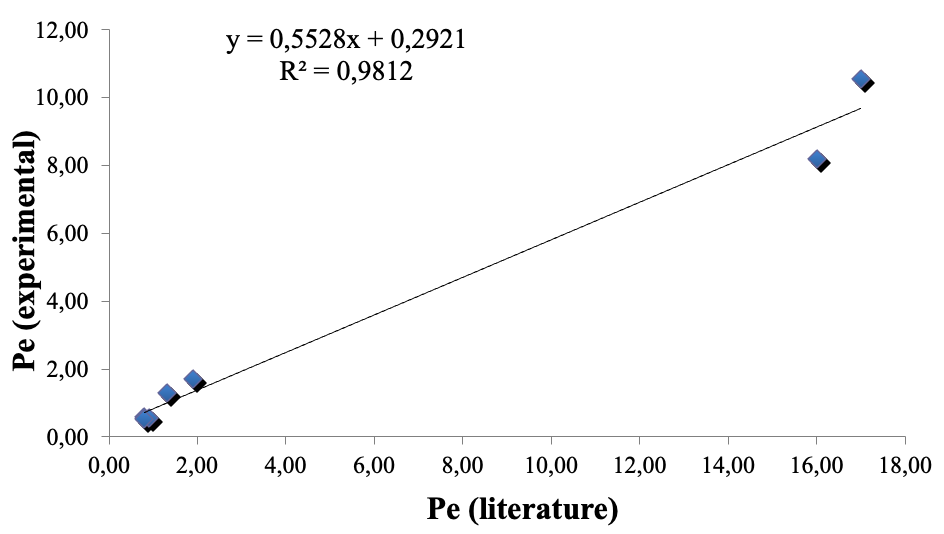


**Table S4.** Permeability coefficient of standard drugs used as control and target compounds by PAMPA-GIT assay.

| **Compounds** | ***Pe** literature**  **(10^-6^ cm s^-1^)** | ***Pe** experimental**  **(10^-6^ cm s^-1^)** | **Fa lit.**  **(%)** | **Fa exp.**  **(%)** | **Classification** |
| --- | --- | --- | --- | --- | --- |
| **Acyclovir** | **0.00** | 0.45 | 2.1 | 1.70 | Low |
| **Atenolol** | **0.10** | 0.10 | 5.2 | 2.26 | Low |
| **Ceftriaxone** | **0.10** | 0.67 | 1 | 1.37 | Low |
| **Coumarin** | **22.90** | 22.78 | 100 | 99.99 | High |
| **Diclofenac** | **12.50** | 13.10 | 100 | 99.31 | High |
| **Hydrocortisone** | **3.40** | 4.77 | 91 | 96.34 | High |
| **Norfloxacin** | **0.90** | 0.40 | 55 | 52.19 | Medium |
| **Ranitidine** | **0.50** | 0.30 | 35 | 38.29 | Medium |
| **Sulfasalazine** | **0.30** | 0.14 | 42 | 44.66 | Medium |
| **Verapamil** | **7.40** | 5.56 | 98 | 93.22 | High |

* Results are expressed by mean (n = 2) of experimental permeability (Pe. Exp, 10-6 cm/s) and absorbed fraction (Fa., %). lit.: literature; exp.: experimental.

**Figure S4.** Linear correlation between experimental and literature permeability (Pe) values. Data represent the mean of triplicates in two independent analyses (n = 2).


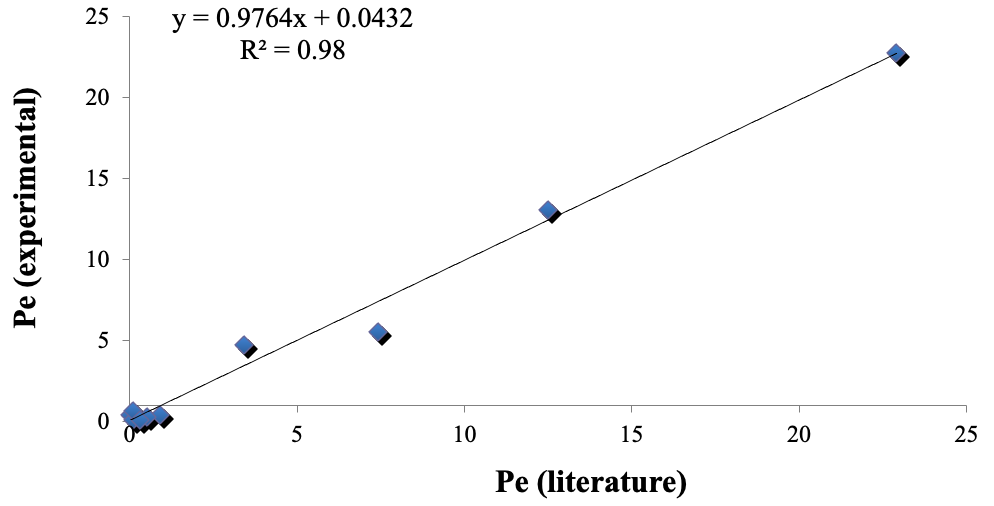


**Figure S5:** A) Liver microsomal stability profile of LASSBio-1971 (**1**); B) First order rate constant (*k*) for elimination. Experiment carried out in the presence of a NADPH generating system.


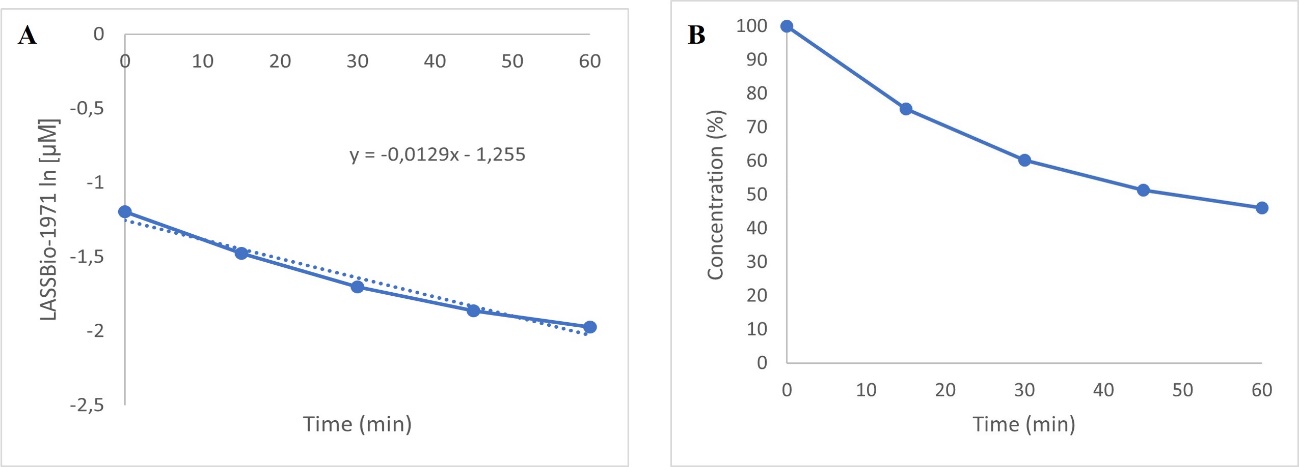


**Figure S6:** A) Liver microsomal stability profile of LASSBio-1971 (**1**); B) First order rate constant (*k*) for elimination. Experiment carried out in the absence of a NADPH generating system.


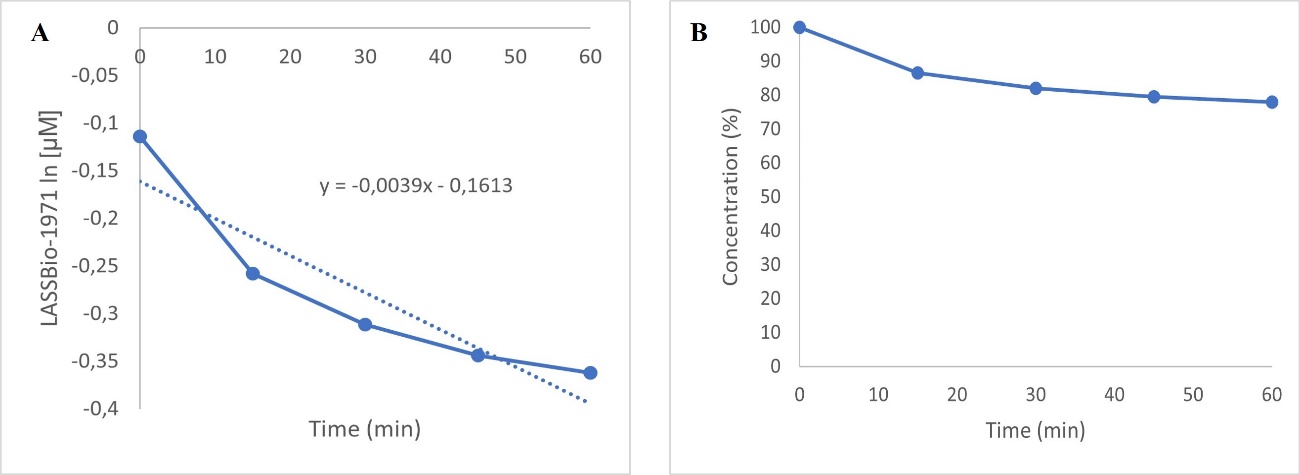


**Figure S7:** A) Liver microsomal stability profile of LASSBio-1974 (**2**); B) First order rate constant (*k*) for elimination. Experiment carried out in the presence of a NADPH generating system.


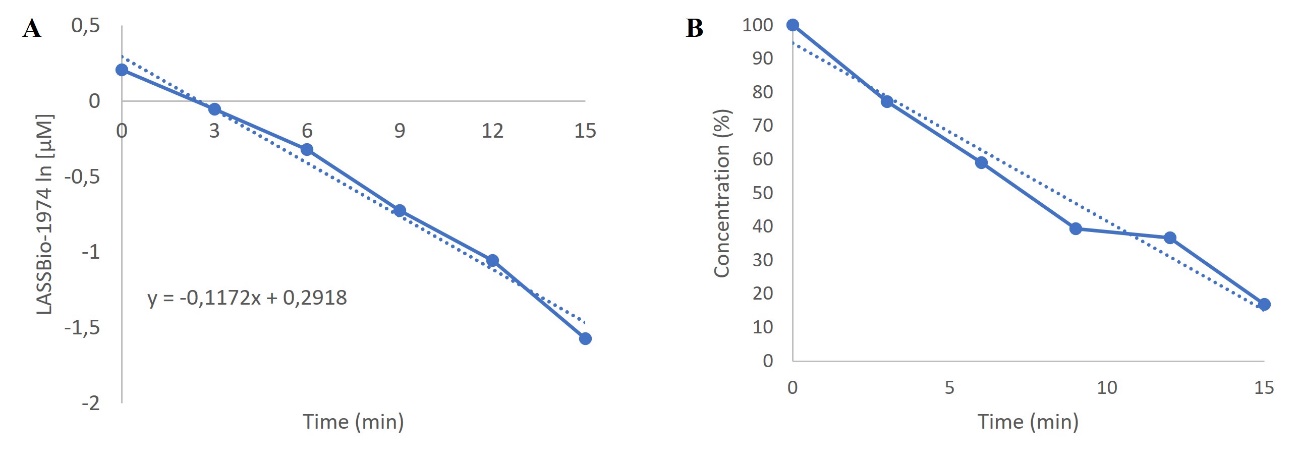


**Figure S8:** A) Liver microsomal stability profile of LASSBio-1974 (**2**); B) First order rate constant (*k*) for elimination. Experiment carried out in the absence of a NADPH generating system.


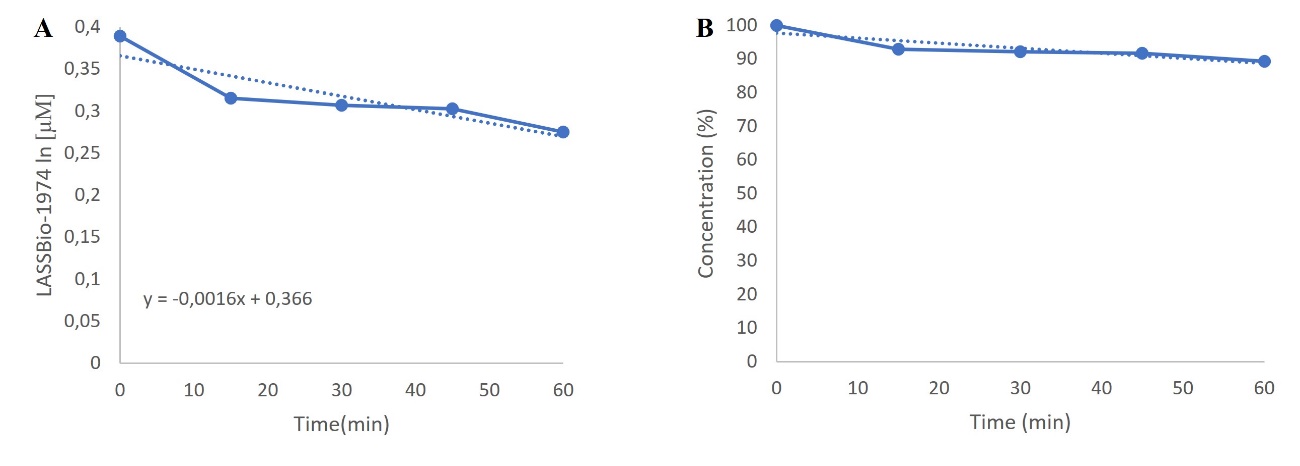


**Figure S9:** LC-ESI(+)-HRMS mass spectra of identification of rat in vitro phase II metabolites of *P*-chlorophenyl isocyanate, used as a standard for method validation; **A**) shows *P*-chlorophenyl isocyanate, the precursor ion of m/z 154.01 [M + H]^+^; and **B**) the fragment obtained from the conjugation product of glutathione of m/z 475.10 [M + H]^+^.


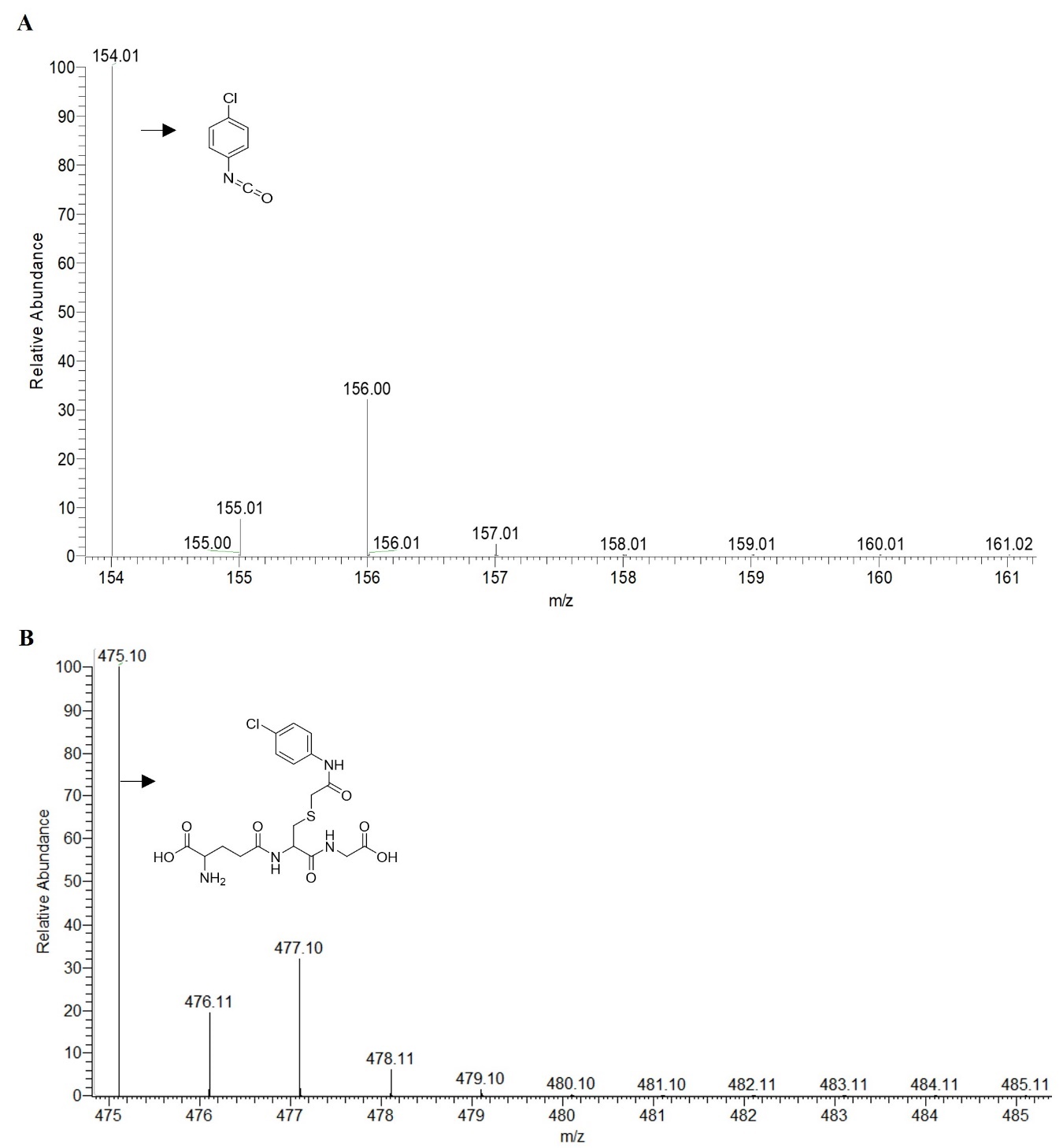


**Figure S10:** LC-ESI(-)-HRMS mass spectra of identification of rat in vitro phase II metabolites of LASSBio-1971 (**1**); **A**) the fragment obtained from the conjugation product of glutathione of m/z 512.17 [M + H]^+^; and **B**) the fragment obtained from the conjugation product of glutathione of m/z 583.21 [M + H]^+^.


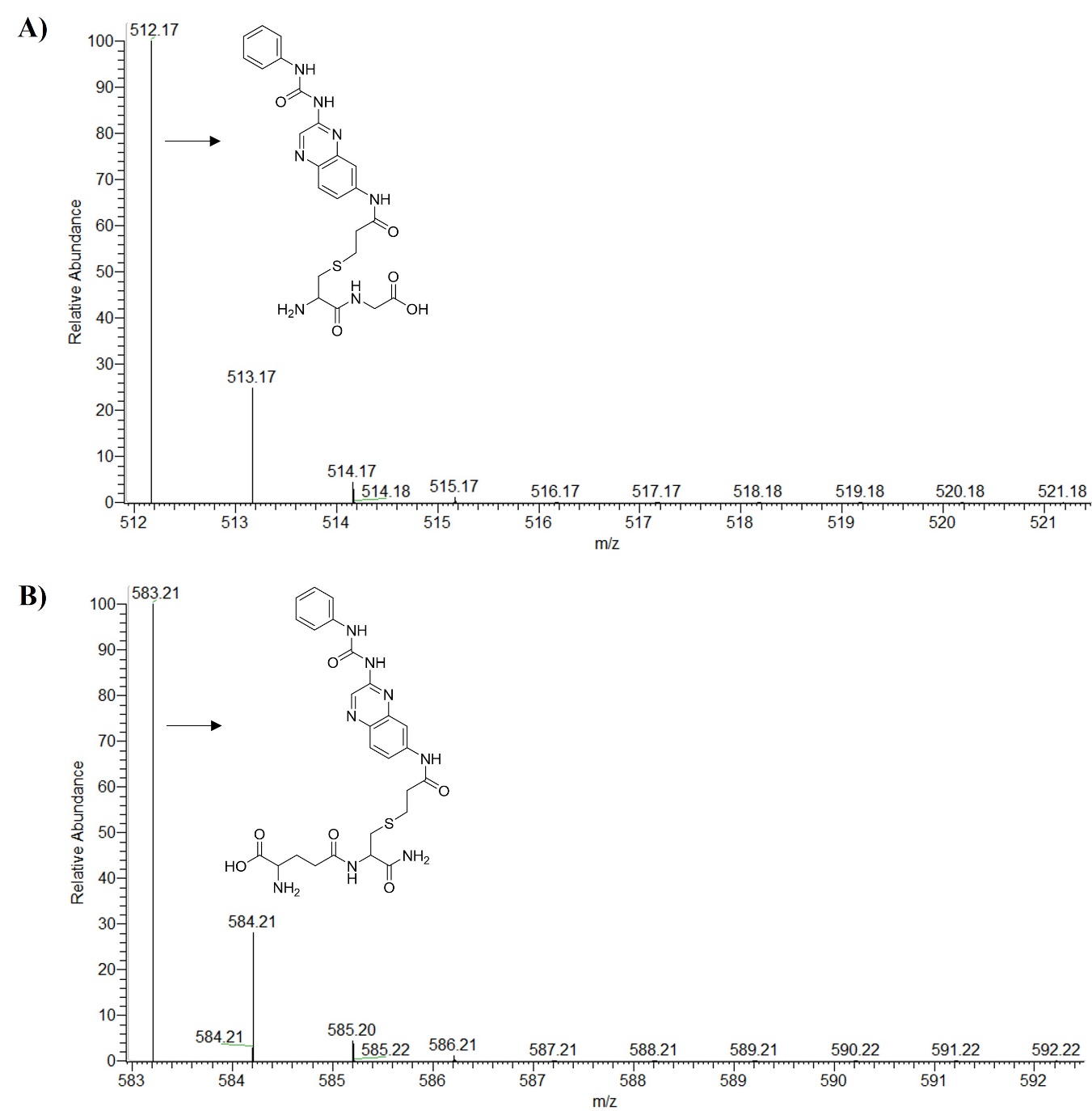

Supplement: Supplementary file 1 — Supp information. Table S1: LASSBio‐1971 (1) and LASSBio‐1974 (2) cytotoxic activity against NSCLC cell line in 48h period assessed by MTT assay. Results are expressed in micromolar (µM) and confidence interval (95%), (n ≥ 3). Table S2: LASSBio‐1971 (1) and LASSBio‐1974 (2) cytotoxic activity against NSCLC cell line in 24h period assessed by MTT assay. Results are expressed in micromolar (µM) and confidence interval (95%), (n ≥ 3). Table S3: Permeability coefficient of standard drugs, used as control and of target compounds by the PAMPA‐BBB technique. Table S4: Permeability coefficient of standard drugs, used as control and of target compounds by the PAMPA‐GIT technique. Figure S1: Cytotoxicity curves of compounds LASSBio‐1971 (1) and LASSBio‐1974 (2) against NSCLC cell lines in a 72h period assessed by (A) MTT assay and (B) SRB assay. Figure S2: Cytotoxicity curves of compounds LASSBio‐1971 (1) and LASSBio‐1974 (2) against NSCLC cell lines assessed by MTT assay in a (A) 48h and (B) 24h period. Figure S3: Linear correlation between experimental and literature permeability (Pe) values. Data represent the mean of triplicates in two different analyses (n = 2). Figure S4: Linear correlation between experimental and literature permeability (Pe) values. Data represent the mean of triplicates in two different analyses (n = 2). Figure S5: A) Liver microsomal stability profile of LASSBio‐1971 (1); B) First order rate constant (k) for elimination. Experiment carried out in the presence of a NADPH generating system. Figure S6: A) Liver microsomal stability profile of LASSBio‐1971 (1); B) First order rate constant (k) for elimination. Experiment carried out in the absence of a NADPH generating system. Figure S7: A) Liver microsomal stability profile of LASSBio‐1974 (2); B) First order rate constant (k) for elimination. Experiment carried out in the presence of a NADPH generating system. Figure S8: A) Liver microsomal stability profile of LASSBio‐1974 (2); B) First order rate co [file ARDP-358-e70151-s001.docx]
